# The efficacy of psychological interventions for anxiety in Parkinson’s disease: a systematic review and meta-analysis including mindfulness and mind–body approaches

**DOI:** 10.1186/s40359-026-05436-y

**Published:** 2026-08-26

**Authors:** Jiyoung Gwak, Jinse Park

**Affiliations:** 1 Graduate School of Counseling, Baekseok University, Seoul, Republic of Korea; 2 Department of Neurology, Haeundae Paik Hospital, Inje University, Busan, Republic of Korea

**Keywords:** Parkinson’s disease, Anxiety, Psychological interventions, Mindfulness, Mind–body interventions, Systematic review, Meta-analysis

## Abstract

**Background:**

Anxiety is a prevalent and clinically significant non-motor symptom in individuals with Parkinson’s disease (PD), yet the overall efficacy of psychological interventions for anxiety in this population remains unclear. This meta-analysis aimed to evaluate the effectiveness of psychological and mind–body interventions in reducing anxiety symptoms among individuals with PD and to explore potential differences according to theoretical orientation.

**Methods:**

Randomized controlled trials (RCTs) examining psychological or mind–body interventions reporting anxiety outcomes in individuals with PD were systematically identified in four databases searched to 27 July 2026. Effect sizes were calculated as standardized mean differences using Hedges’ g within a random-effects model, with between-study variance estimated by restricted maximum likelihood and inference based on the Hartung–Knapp adjustment. Heterogeneity was assessed using Cochran’s Q and I² statistics, and a prediction interval was calculated. Subgroup analyses compared cognitive–behavioral and interpersonal therapies with mindfulness- and mind–body-based approaches, and active with inactive control conditions. Sensitivity analyses, including leave-one-out procedures and exclusion of trials at high risk of bias, were performed. Publication bias was examined using funnel plot inspection and Egger’s regression test, and the certainty of evidence with GRADE.

**Results:**

Across 13 comparisons from 13 trials and 877 participants, psychological and mind–body interventions were associated with a statistically significant reduction in anxiety symptoms (Hedges’ g = − 0.46, 95% CI [− 0.67, − 0.25]). Heterogeneity was moderate (I² = 40.7%, 95% CI 0% to 79%), and the 95% prediction interval, − 1.00 to + 0.08, included the null. Cognitive–behavioral and interpersonal therapies showed a significant effect (g = − 0.57), whereas the estimate for mindfulness- and mind–body-based approaches was smaller and its interval included the null (g = − 0.25); there was no statistical evidence of subgroup differences. Subgroup findings should be interpreted cautiously because of the small number of comparisons within each category. Eleven of the thirteen comparisons used an inactive control condition (g = − 0.53), and the estimate against active comparators was imprecise (g = − 0.22). A similar reduction was observed at follow-up in nine trials (g = − 0.39). Sensitivity analyses confirmed the stability of the pooled effect, and no clear evidence of publication bias was detected.

**Conclusions:**

Psychological and mind–body interventions may reduce anxiety symptoms in individuals with PD, and a similar reduction was observed at follow-up. However, the clinical interpretation of this effect requires caution because different anxiety scales were used across trials, the number of included studies was limited, several studies were rated as having a high risk of bias, the prediction interval included the null, and the effect derives principally from comparisons with inactive control conditions. The overall certainty of the evidence was very low. Although no statistically significant differences between theoretical approaches were observed, this subgroup finding should be interpreted as exploratory, and larger trials recruiting participants with clinically significant anxiety, using active comparators and standardized follow-up, are needed before firm clinical recommendations can be made.

**Supplementary Information:**

The online version contains supplementary material available at https://doi.org/10.1186/s40359-026-05436-y.

## Background

Parkinson’s disease (PD) has traditionally been characterized by its motor symptoms, including bradykinesia, rigidity, tremor, and postural instability. However, increasing attention has been directed toward non-motor symptoms, which are now recognized as key determinants of disease burden and prognosis. Among these, anxiety is one of the most prevalent and clinically consequential symptoms. The prevalence of clinically significant anxiety symptoms in individuals with PD has been estimated at approximately 20–40%, substantially exceeding rates observed in the general older population [1].

Anxiety in PD is best understood as a dimensional symptom construct that may occur with or without a diagnosable anxiety disorder, and that may be shaped by disease-specific neurobiological changes and functional limitations [2]. Anxiety frequently precedes motor onset, fluctuates with dopaminergic state, and has been linked to degeneration in noradrenergic and serotonergic systems rather than to dopaminergic loss alone [3]. The unpredictability of motor fluctuations, progressive loss of bodily control, and the realistic threat of falling can induce persistent vigilance and anticipatory anxiety. Fear of falling, in particular, is frequently observed in PD and represents a distinct manifestation of anxiety that may increase muscular tension and contribute to rigid gait patterns, thereby paradoxically elevating fall risk [4]. This cyclical interaction between avoidance, functional decline, and escalating anxiety suggests that anxiety in PD should be conceptualized as both an emotional symptom in its own right and a central therapeutic target, with potential implications for disease progression and functional outcomes [5].

This conceptualization has a methodological consequence that we state explicitly. Distinct diagnostic criteria for anxiety in PD have not been established, and randomized trials in this field accordingly measure anxiety as symptom severity on validated general anxiety scales rather than as a categorical diagnosis. Anxiety was therefore operationalized in the present review as symptom severity measured by a validated anxiety instrument, which is the outcome the included trials report. Disease-specific presentations such as fear of falling provide interpretive context for what these instruments capture in this population, but were not themselves eligible outcomes.

Pharmacological treatment in PD primarily targets motor symptoms through dopaminergic modulation, and its effects on emotional symptoms such as anxiety are often limited. Although anxiolytics or antidepressants may be prescribed, concerns regarding adverse effects and increased fall risk in older adults complicate pharmacological management [6]. In this context, psychological interventions have emerged as increasingly important non-pharmacological treatment strategies. Cognitive behavioral therapy (CBT) is the most extensively studied psychological intervention in PD and aims to alleviate anxiety by modifying maladaptive cognitions and reducing avoidance behaviors [7]. Several randomized controlled trials have demonstrated significant reductions in anxiety symptoms following CBT in individuals with PD [8].

Nevertheless, the nature of anxiety in PD raises important theoretical considerations. The threats experienced by patients—such as fall risk, motor fluctuations, and progressive functional impairment—are often realistic and persistent rather than purely cognitive distortions. Approaches that conceptualize anxiety solely as a target for cognitive control may therefore have inherent limitations. Mindfulness- and mind–body interventions, which have gained increasing attention, focus not on eliminating uncontrollable bodily sensations or emotional experiences but on altering individuals’ relationship to these experiences and fostering acceptance-oriented coping [9]. Such frameworks may hold particular theoretical relevance in PD, a condition characterized by chronicity and unpredictability.

This distinction provides a principled reason to examine the two families of intervention separately, but not a reason to expect either to be superior overall. Classic stress-and-coping theory holds that the adaptiveness of a coping strategy depends on the controllability of the stressor [10], and PD presents controllable and uncontrollable stressors simultaneously: anticipatory worry about social situations is amenable to cognitive and behavioral change, whereas progressive motor decline is not. To our knowledge, however, no randomized trial has directly compared these two families of intervention for anxiety in PD, and the present review accordingly examines them as parallel subgroups rather than as competing treatments.

Several systematic reviews and meta-analyses have examined the efficacy of psychological interventions in individuals with PD [11]. However, important limitations remain regarding scope and focus.

Previous meta-analyses have examined psychological or non-pharmacological interventions for depression and/or anxiety symptoms in Parkinson’s disease, but their scopes have differed from the present review. Troeung et al. (2013) synthesized randomized placebo-controlled trials for depression and/or anxiety in PD broadly [12]. More recent CBT-focused meta-analyses, including Yu et al. (2025), have evaluated cognitive behavioral therapy for anxiety and depressive symptoms in PD, including remote and face-to-face CBT modalities [13]. However, these reviews primarily focused on CBT or combined anxiety with broader mood-related outcomes, and their pooled estimates have differed markedly in magnitude. In contrast, the present review focuses specifically on anxiety outcomes and synthesizes randomized controlled trials across a broader range of psychological and mind–body interventions.

Furthermore, earlier meta-analyses may not fully reflect recently published randomized controlled trials, and several have pooled anxiety together with depression into a single measure of psychological distress. Combining the two outcomes at the review level may obscure the independent clinical significance and functional impact of anxiety in PD, although a trial that assesses both outcomes provides usable anxiety data and is eligible for synthesis on that basis. Consequently, clinicians currently lack integrated evidence to inform decisions regarding which theoretical approaches may be most appropriate in the specific context of PD-related anxiety.

Against this background, the present study aimed to quantitatively synthesize evidence from randomized controlled trials examining psychological and mind–body interventions for anxiety in individuals with PD, with anxiety treated as the primary outcome of the present review; to examine whether a similar reduction was observed at follow-up; and to examine whether it is consistent across intervention type, control condition, outcome instrument, and risk of bias.

Interventions were categorized into cognitive–behavioral and interpersonal therapies and mindfulness- and mind–body-based approaches, and exploratory subgroup analyses were conducted to examine potential differences in effect patterns across theoretical models [14].

Rather than asserting the superiority of any single approach, this study seeks to expand the empirical foundation for understanding disease-specific anxiety—particularly fear of falling and activity avoidance—and to inform the development of tailored intervention strategies. By clarifying the magnitude and pattern of treatment effects, this meta-analysis aims to contribute to more refined psychological interventions designed to enhance functional engagement and quality of life in individuals with PD.

## Methods

### Protocol and reporting guidelines

This study was conducted in accordance with the Preferred Reporting Items for Systematic Reviews and Meta-Analyses (PRISMA) 2020 guidelines, and reporting followed these recommendations [15]. The review protocol and analytical plan were developed before data extraction; however, the review was not registered in PROSPERO. Analyses that were prespecified in that plan and analyses added after the results were known are identified as such throughout.

### Search strategy

A systematic literature search was conducted in PubMed, Embase, and the Cochrane Central Register of Controlled Trials (CENTRAL) on 10 February 2026. The search was subsequently extended to include Web of Science and re-run across all four databases on 27 July 2026, so that a single search date applies to every source. PsycINFO was not searched, as the institution did not hold a subscription to that database, and this limitation is acknowledged in the Discussion. Web of Science was added to broaden the search coverage. Searches were performed from database inception through the respective search dates.

The search strategy was structured around two core concepts: (1) Parkinson’s disease, and (2) psychological, psychosocial, mindfulness, and mind–body interventions.

Controlled vocabulary terms (e.g., MeSH, Emtree) and free-text keywords were used in combination, and Boolean operators (AND, OR) were applied to construct the search queries. To maximize sensitivity, synonyms and spelling variations were included where appropriate, and anxiety-related outcomes and the randomized controlled trial study design were not incorporated as search terms but were instead applied as eligibility criteria during study selection. The initial search retrieved 15,487 records and the updated search retrieved 20,338 records. The reference lists of the included trials and of relevant reviews were additionally screened; forward citation searching was not carried out. The complete search strategies, search dates, and database-specific yields are provided in Supplementary Appendix A. All supplementary appendices and tables are provided in a separate Supplementary Materials file.

### Eligibility criteria

Studies were included if they met the following criteria:

1. adults aged 18 years or older diagnosed with Parkinson’s disease;
2. randomized controlled trials evaluating a psychological or mind–body intervention delivered with an explicit therapeutic rationale as the primary intervention;
3. quantitative assessment of anxiety symptoms using validated measures; and.
4. sufficient statistical information to compute effect sizes, including means and standard deviations or convertible data for comparisons between intervention and control groups

Studies were excluded if they met any of the following criteria:

1. non-randomized or single-arm designs;
2. mixed diagnostic samples without separable PD-specific data;
3. anxiety outcomes not reported independently;
4. only categorical data presented;
5. summary or dispersion statistics were insufficient or could not be converted to an effect size;
6. duplicate data; or
7. protocol-only publications.

Neither clinically significant anxiety at baseline nor anxiety as the primary treatment target was required for inclusion. This was a deliberate decision: a trial that reports usable anxiety data provides evidence about the effect of the intervention on anxiety whether or not anxiety was its principal target, and requiring either condition would have excluded a substantial part of the available evidence. The consequence, which we state explicitly rather than leave to be inferred, is that the synthesis combines trials addressing related but distinct clinical questions. We therefore record for each trial whether anxiety was a designated primary or co-primary outcome and whether elevated anxiety was required at study entry (Table 1), and we examine both characteristics as exploratory subgroups.

Interventions were required to target cognitive, acceptance-based, or mindfulness-based mechanisms explicitly. Interventions whose stated rationale was physical training, movement practice, or positive affect induction without an explicit psychological mechanism — including tai chi, qigong, laughter yoga, and positive psychology programs — were not eligible.

The terminology used in this review is defined as follows. A psychological intervention denotes a structured intervention delivered with an explicit psychological rationale and a stated mechanism of change. A mind–body intervention denotes an intervention in which attentional training, breath regulation, or guided movement serves as the vehicle for that psychological mechanism. The terms psychosocial and behavioral are used only where they appear in the source reports and are not treated as separate categories. Interventions whose stated rationale is physical training alone, without a psychological mechanism, fall outside these definitions and were not eligible; structured exercise and stretching therefore appear in this review only as comparator conditions.

Eligibility was restricted to reports published in English. This restriction was not prespecified but was applied consistently throughout screening because translation resources were unavailable to the review team. Only studies involving human participants were included, and no restriction was placed on publication year.

### Study selection

**Fig. 1:**
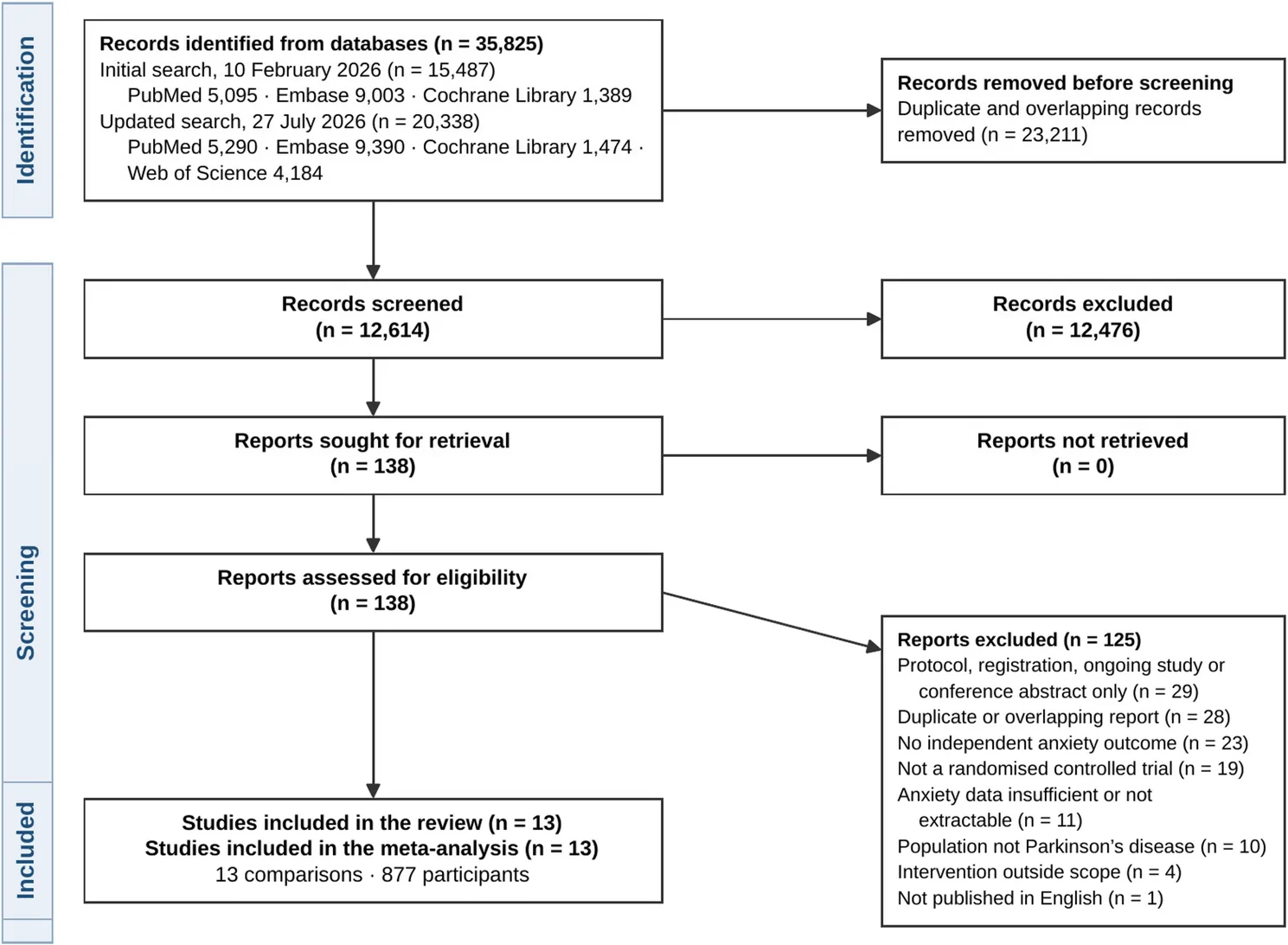
PRISMA 2020 flow diagram

Records from the two searches were combined in EndNote and deduplicated against one another, so that records common to the two searches were counted once. Titles and abstracts were then screened against the eligibility criteria to identify reports requiring full-text assessment; this stage was conducted by a single reviewer. Full-text eligibility assessment was conducted independently by two reviewers, with disagreements resolved through discussion and, when necessary, consultation with a third reviewer. Inter-rater agreement was calculated for the 104 reports with complete paired pre-consensus binary include/exclude judgments (Cohen’s κ = 0.735, 95% CI 0.59–0.88; observed agreement 89.4%). The study selection process is illustrated in the PRISMA 2020 flow diagram (Fig. 1).

### Data extraction

Data extraction was independently conducted by two reviewers, with discrepancies resolved through discussion. When necessary, corresponding authors were contacted for additional information.

The following data were extracted: first author and publication year, country of study, sample size (intervention/control), mean age and proportion of female participants, intervention type and theoretical orientation, intervention duration, control condition, anxiety outcome measures, whether anxiety was a designated primary or co-primary outcome, and whether elevated anxiety was required at study entry. Where a trial reported an assessment after the end of the intervention period, the corresponding follow-up values were extracted if both randomized groups were assessed and the control group had not crossed over to the experimental intervention before that assessment. Follow-up values were extracted in the form reported by the original study, and any conversion from standard errors, confidence intervals, medians or ranges was documented.

For effect size calculation, post-intervention means and standard deviations were used. To minimize unit-of-analysis errors, we examined author groups, study sites, recruitment periods, trial registration information, intervention descriptions, and participant characteristics to identify potentially overlapping samples or duplicate reports. When multiple publications appeared to report data from the same trial or overlapping participant samples, the most complete dataset for post-intervention anxiety outcomes was retained. Where standard errors rather than standard deviations were reported, standard deviations were derived as SE × √n, and where medians and ranges were reported, means and standard deviations were estimated using the method of Wan et al. [16].

For the one three-arm trial, the two active intervention arms were combined at the group-data level using the formula described by Borenstein et al. [17], yielding a single comparison against the shared control group. Alternative specifications, in which the two arms are retained as separate comparisons, are compared with the combined-arm analysis in Supplementary Table S7, together with robust variance estimation and a three-level model.

### Risk of bias assessment

Methodological quality was assessed using the Cochrane Risk of Bias 2 (RoB 2) tool [18]. Evaluation was based on the primary outcome (post-intervention anxiety) and included five domains: (1) the randomization process; (2) deviations from intended interventions; (3) missing outcome data; (4) measurement of the outcome; and (5) selection of the reported result.

Each domain was rated as “low risk,” “some concerns,” or “high risk.” Studies were classified as having overall high risk of bias if at least one domain was rated as high risk. Two reviewers independently conducted the assessments, and disagreements were resolved through discussion. Inter-rater agreement, calculated from the independent judgments recorded before discussion, was substantial at domain level (Cohen’s κ = 0.610, 95% CI 0.41 to 0.81; 81.5% observed agreement; 65 paired judgments) and almost perfect for the overall judgment (κ = 0.871, 95% CI 0.63 to 1.00; 92.3% observed agreement); disagreements were most frequent in the domains concerning measurement of the outcome and the randomization process. The certainty of the evidence was assessed with the GRADE approach by two reviewers independently [19], and a Summary of Findings table is provided (Table 2).

### Statistical analysis

Effect sizes were calculated as standardized mean differences using Hedges’ g, with small-sample bias correction applied [20].

Because anxiety symptoms were assessed using different validated instruments across studies, standardized mean differences were used to place treatment effects on a common metric. A random-effects model was selected a priori because clinical and methodological heterogeneity was expected across studies, including differences in intervention type, delivery format, control condition, and anxiety outcome measures [21]. Between-study variance was estimated by restricted maximum likelihood and confidence intervals were derived using the Hartung–Knapp adjustment. The DerSimonian–Laird and Paule–Mandel estimates are reported alongside the primary model (Supplementary Table S3).

Statistical heterogeneity was assessed using Cochran’s Q statistic and the I² index [22]. I² values of 25%, 50%, and 75% were interpreted as indicating low, moderate, and high heterogeneity, respectively. A 95% confidence interval for τ² was obtained by the Q-profile method, and a 95% prediction interval was calculated to describe the range of effects expected in a future comparable trial.

Two intervention families were distinguished for subgroup analysis by their stated mechanism of change. Cognitive–behavioral and interpersonal therapies denote protocol-based talking therapies in which change is sought by identifying and modifying the appraisals, behaviors, or interpersonal patterns that maintain distress; this family comprises cognitive behavioral therapy and interpersonal psychotherapy. Mindfulness- and mind–body-based approaches denote interventions in which attentional training, breath regulation, and nonjudgmental awareness are the stated mechanism, and in which the aim is to alter a person’s relationship to distressing experience rather than its content; this family comprises mindfulness training, meditation, and mindfulness yoga. Each family is therefore defined by the mechanism it invokes rather than by the mechanism it does not. We note that cognitive control is not the primary mechanism of interpersonal psychotherapy, and the subgroup is therefore labeled by the interventions it contains rather than by an assumed common mechanism. Between-group differences were examined using the Q-between statistic.

Two sensitivity analyses were prespecified in the original analytical plan: a leave-one-out procedure, now conducted at trial level, and exclusion of studies rated as high risk of bias [23, 24]. All remaining analyses reported below were conducted after the results were known and are identified as exploratory. These comprised stratification by control condition, distinguishing inactive conditions from active comparators and additionally examining the inactive conditions in three strata; stratification by whether anxiety was a designated primary or co-primary outcome and by whether elevated anxiety was required at study entry; three analyses addressing the pooling of different anxiety instruments, namely comparison of clinician-administered with self-report instruments, exclusion of the single trial using the trait subscale of the State–Trait Anxiety Inventory, and comparison of instruments with a high versus low proportion of somatic items; meta-regression on mean age and the proportion of female participants; restriction to trials at low risk of bias; exclusion of trials with fewer than 20 participants per arm; and exclusion of trials for which standard deviations had been derived by conversion or estimation.

Publication bias was evaluated through visual inspection of funnel plot asymmetry, Egger’s regression test [25], the rank correlation test, and the trim-and-fill procedure. Given the limited number of included studies, publication bias results were interpreted cautiously. Statistical significance was set at *p* < .05.

All analyses were performed in Python 3.12.3 using NumPy 2.4.4, SciPy 1.17.1 and openpyxl 3.1.5. Hedges’ g and its sampling variance were calculated from group-level summary statistics with a small-sample correction. Random-effects models were fitted by restricted maximum likelihood estimation of the between-study variance, implemented by numerical minimization of the restricted likelihood using scipy.optimize.minimize_scalar. Hartung–Knapp-adjusted confidence intervals and two-sided P values were obtained from the t distribution in scipy.stats.t, and the confidence interval for τ² was obtained by the Q-profile method using scipy.optimize.brentq. Cochran’s Q, I², prediction intervals, subgroup comparisons, meta-regression, leave-one-out analyses and tests for publication bias were computed within the same analysis script. Extracted data and analytical outputs were checked against the source reports and calculation procedures by the authors. The analytical dataset and the complete analysis script are openly available at the Open Science Framework (https://doi.org/10.17605/OSF.IO/6G84B).

## Results

### Study selection

A total of 35,825 records were identified through database searches. After duplicate and overlapping records were removed, 12,614 records remained for title and abstract screening, of which 12,476 were excluded. The remaining 138 reports were sought for retrieval; all were retrieved and assessed for eligibility.

Of the 138 reports assessed for eligibility, 125 were excluded. The most frequent reasons were that the report was a protocol, registration record, ongoing study record or conference abstract (*n* = 29); that it was a duplicate or overlapping report of an included trial (*n* = 28); that no independent anxiety outcome was reported (*n* = 23); that the design was not a randomized controlled trial (*n* = 19); that anxiety data were insufficient or not extractable (*n* = 11); that the population was not Parkinson’s disease (*n* = 10); that the intervention fell outside the scope of the review (*n* = 4); and that the report was not published in English (*n* = 1). Selected reports requiring individual explanation after initial eligibility assessment are listed with reasons for non-inclusion in Supplementary Table S1.

Thirteen randomized controlled trials were included in the qualitative synthesis, and the same thirteen trials were included in the quantitative synthesis. Because the two active arms of the one three-arm trial were combined at the group-data level, each trial contributes exactly one comparison, giving thirteen comparisons in the meta-analysis. The study selection process is presented in the PRISMA 2020 flow diagram (Fig. 1).

### Study characteristics

The characteristics of the thirteen included trials are presented in Table 1. The studies were conducted across eight countries and one special administrative region (the United States, the United Kingdom, Australia, Canada, Sweden, Germany, and the Netherlands and France jointly, together with Hong Kong), encompassing both Western and Asian settings. A total of 877 participants contributed to the post-treatment analyses, 472 in intervention groups and 405 in control groups, with individual trial sizes ranging from 11 to 159. The mean age of participants ranged from 58.8 to 68.8 years, indicating that most trials enrolled middle-aged to older adults with Parkinson’s disease, and the proportion of female participants ranged from 25.0% to 61.0%. Intervention duration was relatively consistent: six trials delivered ten weeks of intervention, five delivered eight weeks, and two delivered twelve sessions.

**Table 1:** Characteristics of included randomized controlled trials

Study (Year)	Country	Sample size (Int. / Ctrl)	Mean age (years)	% Female	Intervention mode	Theoretical model	Duration	Control condition	Anxiety measure	Anxiety a primary outcome	Numerical anxiety-scale cut-off required at entry
Bogosian et al. (2022) [26]	United Kingdom	30 / 30	60.9	50.0	Videoconferencing mindfulness (MBCT-based)	Mindfulness / mind–body	8 weeks	Waitlist	HADS-A	Yes	No
Buchwitz et al. (2021) [27]	Germany	14 / 16	61.0	40.0	Mindfulness training (IPSUM)	Mindfulness / mind–body	10 weeks	Waitlist	STAI-T	No	No
Dobkin et al. (2011) [28]	United States	41 / 39	64.6	40.0	PD-specific CBT	Cognitive–behavioral / interpersonal	10 weeks	CMO	HAM-A	No	No
Dobkin et al. (2020) [29]	United States	37 / 35	65.2	51.4	Telephone CBT	Cognitive–behavioral / interpersonal	10 weeks	TAU	HAM-A	No	No
Dobkin et al. (2021) [30]	United States	45 / 45	64.1	44.4	Video-to-home CBT	Cognitive–behavioral / interpersonal	10 weeks	TAU	HAM-A	No	No
Koszycki et al. (2025) [31]	Canada	32 / 31	64.8	25.0	Interpersonal psychotherapy	Cognitive–behavioral / interpersonal	12 weeks	Supportive therapy	BAI	No	No
Kraepelien et al. (2020) [32]	Sweden	38 / 39	66.0	61.0	Internet-based CBT	Cognitive–behavioral / interpersonal	10 weeks	Waitlist	HADS-A	No	No
Kwok et al. (2019) [33]	Hong Kong	71 / 67	63.6	52.9	Mindfulness yoga	Mindfulness / mind–body	8 weeks	Stretching / resistance	HADS-A	Yes	No
Kwok et al. (2025) [34]	Hong Kong	105 / 54	63.7	47.1	Meditation and yoga (arms combined)	Mindfulness / mind–body	8 weeks	Usual care / waitlist	HADS-A	Yes	No
Moonen et al. (2021) [8]	Netherlands / France	24 / 24	63.3	52.1	PD-specific CBT	Cognitive–behavioral / interpersonal	10 weeks	CMO	HAM-A	Yes	Yes
Okai et al. (2013)[35]	United Kingdom	22 / 13	58.8	31.1	Nurse-delivered CBT for ICB	Cognitive–behavioral / interpersonal	12 sessions	Waiting list + SMC	BAI	No	No
Troeung et al. (2014) [36]	Australia	7 / 7	66.0	33.3	Group CBT	Cognitive–behavioral / interpersonal	8 weeks	Waitlist	DASS-A	Yes	No
Wuthrich & Rapee (2019) [37]	Australia	6 / 5	68.8	36.4	Telephone CBT	Cognitive–behavioral / interpersonal	8 weeks	Waitlist	GAI	Not designated	No

Trials are ordered alphabetically by first author. Theoretical model denotes the subgroup classification used in the analysis: cognitive–behavioral and interpersonal therapies, and mindfulness- and mind–body-based approaches. Anxiety a primary outcome indicates that anxiety was a designated primary or co-primary outcome of the trial; “Not designated” indicates that the report stated no outcome hierarchy. A numerical anxiety-scale cut-off at entry was required in one trial only. Sample sizes correspond to the analyzed samples used for effect size calculation. For Kwok et al. (2025), the meditation and yoga arms were combined at the group-data level to avoid a unit-of-analysis error arising from the shared control group. Mean age and percentage female are based on the full randomized sample (Okai et al., 2013: 45 randomized, 35 analyzed).

*BAI*  Beck Anxiety Inventory, *CBT*  cognitive behavioral therapy, *CMO*  clinical monitoring only, *DASS-A*  Depression Anxiety Stress Scales–Anxiety subscale, *GAI*  Geriatric Anxiety Inventory, *HADS-A*  Hospital Anxiety and Depression Scale–Anxiety subscale, *HAM-A*  Hamilton Anxiety Rating Scale, *ICB*  impulse control behaviors, *MBCT*  mindfulness-based cognitive therapy, *PD*  Parkinson’s disease, *SMC* standard medical care, *STAI-T*  State–Trait Anxiety Inventory–Trait subscale, *TAU*  treatment as usual

In terms of theoretical orientation, nine trials evaluated cognitive–behavioral or interpersonal therapies and four evaluated mindfulness- or mind–body-based approaches. Delivery formats varied and included face-to-face, telephone-delivered, internet-delivered, videoconferencing and nurse-delivered cognitive behavioral therapy, interpersonal psychotherapy, mindfulness training, meditation and mindfulness yoga.

Control conditions consisted of a waiting list or usual care in seven trials, clinical monitoring without new treatment in two, treatment as usual in two, and an active comparator in two, the latter comprising a structured stretching and resistance exercise program and supportive therapy. Anxiety outcomes were assessed using standardized measures: the Hamilton Anxiety Rating Scale in four trials, the anxiety subscale of the Hospital Anxiety and Depression Scale in four, the Beck Anxiety Inventory in two, and the Geriatric Anxiety Inventory, the anxiety subscale of the Depression Anxiety Stress Scales, and the trait subscale of the State–Trait Anxiety Inventory in one trial each. Anxiety was a designated primary or co-primary outcome in five trials and a secondary outcome in seven, one trial stated no outcome hierarchy, and one trial required a numerical cut-off on an anxiety scale at study entry.

### Risk of bias

**Fig. 2:**
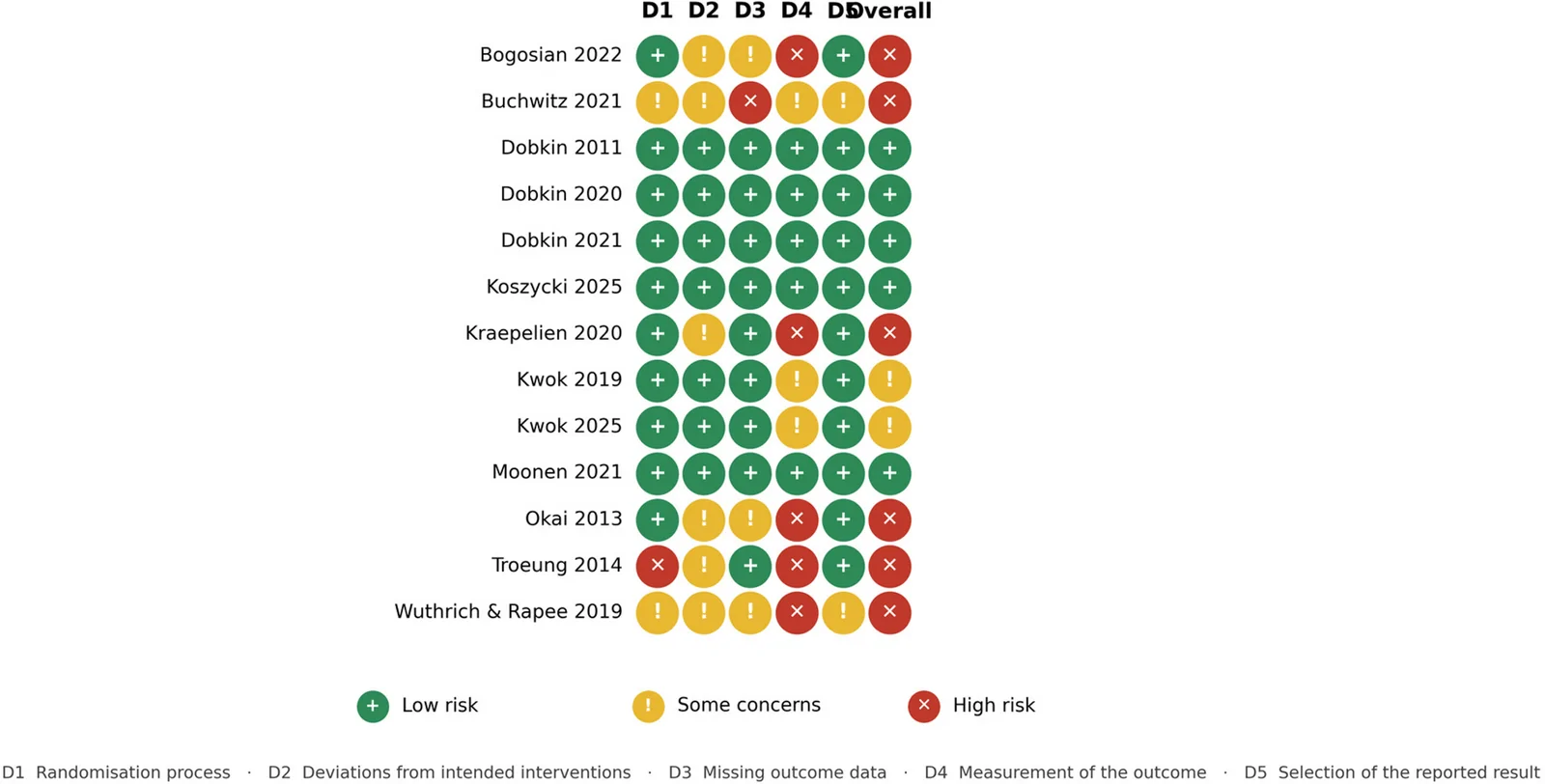
Risk-of-bias assessment for the thirteen included trials (Cochrane Risk of Bias 2). Trials are ordered alphabetically by first author. D1 randomization process; D2 deviations from intended interventions; D3 missing outcome data; D4 measurement of the outcome; D5 selection of the reported result

The risk of bias of the thirteen included trials was assessed using the Cochrane Risk of Bias 2 tool, and the detailed results are presented in Fig. 2.

Regarding the randomization process, several trials were rated as having some concerns or high risk owing to insufficient reporting of allocation concealment. Given the nature of psychological interventions, blinding of participants and intervention providers was generally not feasible, which increased the potential for performance bias. The domain most frequently rated as high risk was measurement of the outcome: trials that assessed anxiety with a self-report instrument in unblinded participants allocated to a waiting-list condition were rated at high risk in this domain. With respect to missing outcome data, most trials clearly reported attrition and applied intention-to-treat analyses, and the risk of selective reporting was generally rated as low risk or as having some concerns.

Overall, the included trials showed a mixed distribution of risk-of-bias ratings: five trials were classified as low risk, two as having some concerns, and six as high risk.

### GRADE working group grades of evidence

High certainty: we are very confident that the true effect lies close to that of the estimate of the effect. Moderate certainty: we are moderately confident in the effect estimate; the true effect is likely to be close to the estimate, but there is a possibility that it is substantially different. Low certainty: our confidence in the effect estimate is limited; the true effect may be substantially different from the estimate. Very low certainty: we have very little confidence in the effect estimate; the true effect is likely to be substantially different from the estimate.

### Explanations

- Downgraded one level for risk of bias. Blinding of participants and providers is not feasible for psychological interventions and most anxiety outcomes were self-reported; several trials also reported allocation concealment incompletely.
- Downgraded one level for indirectness. Most trials did not require clinically elevated anxiety at study entry, anxiety was a secondary outcome in seven of the thirteen trials, and eleven of the thirteen comparisons used an inactive control condition, so the evidence bears less directly on the treatment of clinically significant anxiety and does not separate specific from non-specific effects. For the follow-up outcome this judgment also reflects the unstandardized follow-up intervals (8 weeks to 6 months) and the monthly booster sessions permitted in two trials.
- Rated down one level for serious inconsistency. Moderate between-study heterogeneity was observed (I² = 40.7%, 95% CI approximately 0% to 79%), the 95% prediction interval included the null (− 1.00 to + 0.08), and one eligible trial estimated an effect in the opposite direction. Although heterogeneity was largely attributable to that trial in sensitivity analysis, the variability could not be regarded as sufficiently explained to avoid rating down. Inconsistency was assessed separately for each analysis in this table: the same judgment was applied to the inactive-control analysis, in which the pattern of variability was comparable (I² = 35.5%; prediction interval − 1.09 to + 0.03), whereas no rating down for inconsistency was applied to the active-comparator analysis (I² = 13.1%) or to the follow-up analysis (I² = 26.0%, with substantial overlap of trial-level confidence intervals and a narrower dispersion of effect estimates than in the primary analysis).
- The nine trials contributing a follow-up estimate are not a random subset of the evidence base; the estimate should therefore not be compared directly with the estimate for all thirteen trials.
- Downgraded one further level for imprecision. Only two trials used an active comparator, and the confidence interval spans values indicating substantial benefit, no effect and harm.

Effect estimates are standardized mean differences (Hedges’ g) calculated from post-treatment group means and standard deviations, with negative values indicating lower anxiety in the intervention group. Estimates were pooled using a random-effects model with restricted maximum likelihood estimation and the Hartung–Knapp adjustment. Because six different instruments were used, effects are not expressed on a single raw-score scale.

### Overall effect

Across thirteen comparisons, the random-effects meta-analysis showed a statistically significant reduction in anxiety symptoms associated with psychological and mind–body interventions (Hedges’ g = − 0.46, 95% CI [− 0.67, − 0.25], t = − 4.84, df = 12, *p* = .0004). This effect represents a standardized reduction of moderate statistical magnitude and does not correspond to a single raw-score change, because six different anxiety scales were used across trials.

Between-study heterogeneity was moderate (τ² = 0.052, 95% CI 0.000 to 0.238; I² = 40.7%, 95% CI 0% to 79%; Q = 20.25, df = 12, *p* = .062). The confidence intervals for both τ² and I² include zero, so the magnitude of between-study variance is estimated imprecisely and should not be over-interpreted in either direction. Estimating the between-study variance by the Paule–Mandel method rather than restricted maximum likelihood gave τ² = 0.044 and an identical pooled estimate (g = − 0.46, 95% CI [− 0.67, − 0.25]).

**Fig. 3:**
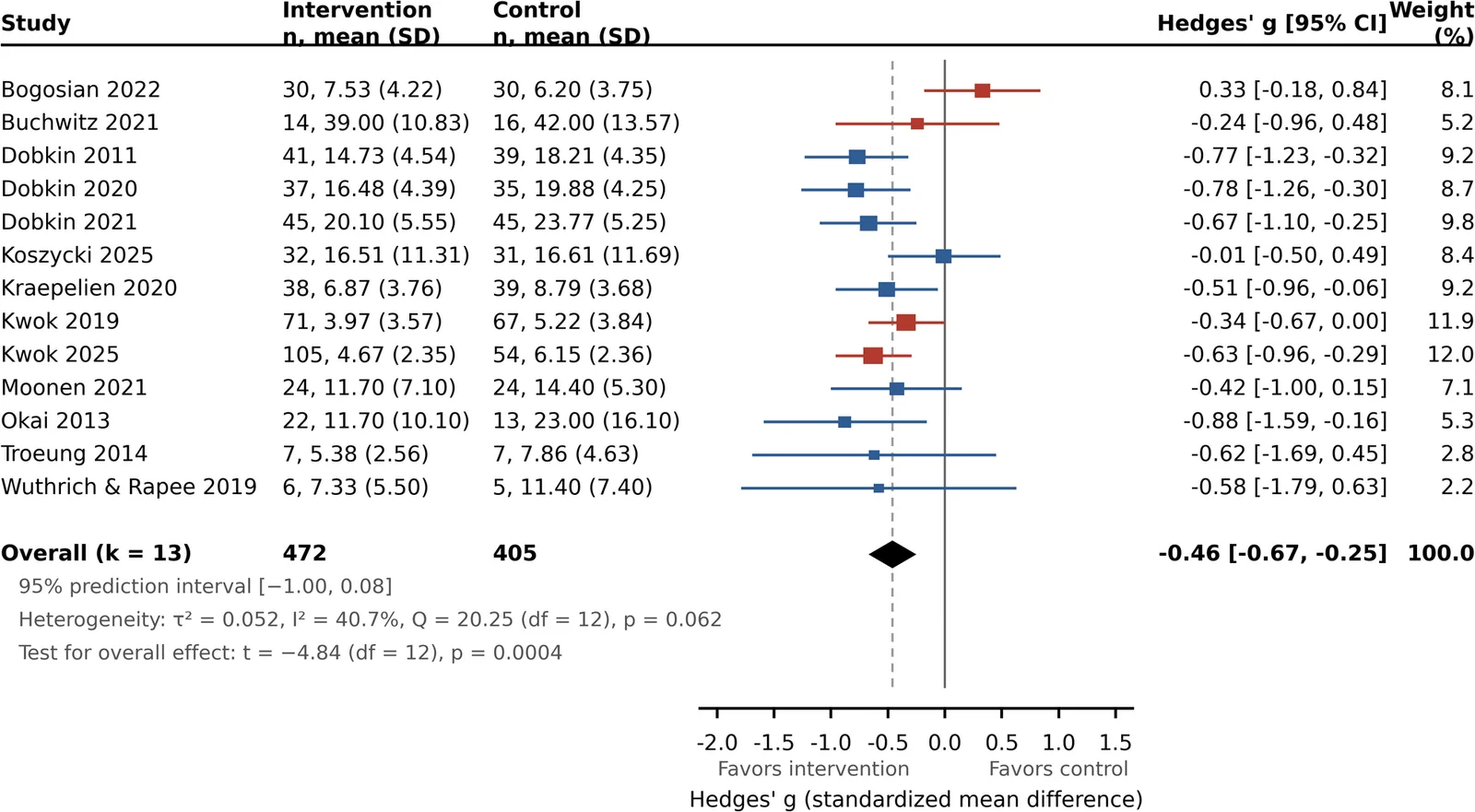
Forest plot of the thirteen included comparisons. Trials are ordered alphabetically by first author. Estimates are Hedges’ g with 95% confidence intervals; the pooled estimate uses restricted maximum likelihood estimation with the Hartung–Knapp adjustment

The 95% prediction interval extended from − 1.00 to + 0.08 and therefore included the null, indicating that the effect observed in a future trial drawn from the same distribution could range from large and favorable to absent. Inspection of individual trial estimates showed that twelve of the thirteen comparisons favored the intervention group (Fig. 3); the single exception was Bogosian et al. (2022), in which anxiety scores were slightly higher in the intervention arm at post-treatment (g = + 0.33).

### Effect at follow-up

Nine trials provided an assessment after the end of the intervention period at which both randomized groups were assessed and the control group had not crossed over to the experimental intervention, contributing 708 participants; follow-up intervals ranged from 8 weeks to 6 months. Pooled under the same specification used for the primary analysis, the effect at follow-up was g = − 0.39 (95% CI [− 0.60, − 0.18]; I² = 26.0%). Restricting the post-treatment analysis to these same nine trials gave g = − 0.41 (95% CI [− 0.70, − 0.13]), so that the within-set comparison is − 0.41 at the end of treatment against − 0.39 at follow-up. The two estimates were not compared statistically.

The estimate was stable across specifications. Excluding the single trial whose follow-up interval was 8 weeks left it unchanged (k = 8, g = − 0.39, 95% CI [− 0.62, − 0.15]), as did restriction to the four trials with a 6-month follow-up (g = − 0.38, 95% CI [− 0.75, − 0.02]) and exclusion of the trial for which approximate standard deviations were derived from model-based confidence intervals (k = 8, g = − 0.43, 95% CI [− 0.67, − 0.19]). Two considerations qualify the interpretation of these estimates. Follow-up intervals were not standardized, and in two trials monthly booster sessions were permitted in the intervention arm during an extension phase, so the estimates represent effects observed at post-treatment follow-up or during an extension phase rather than maintenance after complete treatment cessation. The four trials that could not contribute a follow-up estimate were excluded for structural reasons: in two, the waiting-list condition received the intervention immediately after the post-treatment assessment; in one, the six-month assessment was the trial endpoint rather than a follow-up; and in one, follow-up values were not reported for both groups. Trial-level follow-up estimates, with the summary statistics reported by each trial, are given in Supplementary Table S6.

### Subgroup analysis by intervention type

**Fig. 4:**
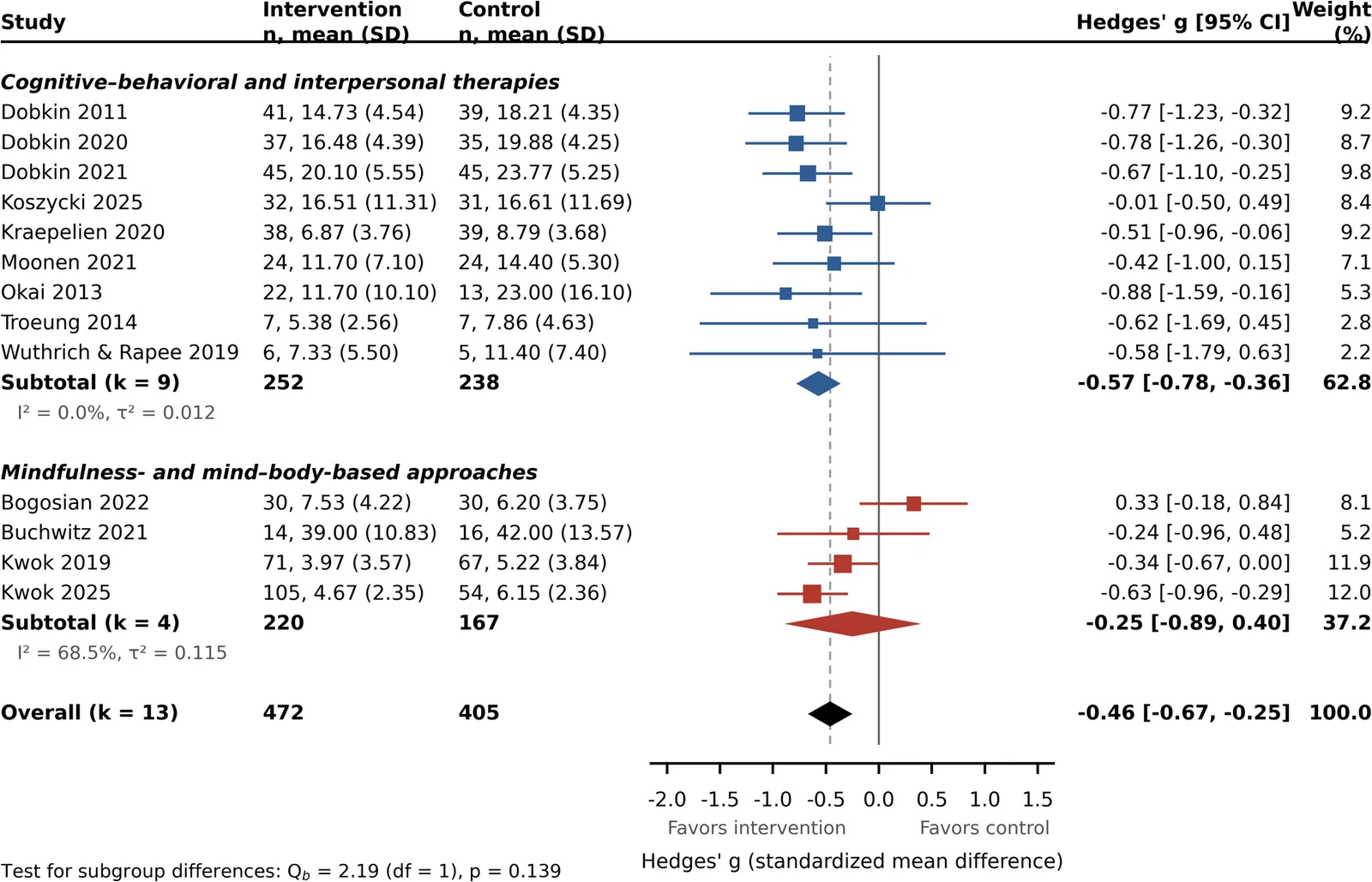
Forest plot by intervention type. Trials are ordered alphabetically by first author within each subgroup, since the subgroup structure takes precedence over the overall ordering

Subgroup analyses were conducted according to theoretical orientation. Cognitive–behavioral and interpersonal therapies showed a statistically significant reduction in anxiety symptoms with no detectable heterogeneity (g = − 0.57, 95% CI [− 0.78, − 0.36], k = 9, *n* = 490, I² = 0%). Mindfulness- and mind–body-based approaches showed a smaller pooled estimate whose confidence interval included the null, with substantial heterogeneity (g = − 0.25, 95% CI [− 0.89, 0.40], k = 4, *n* = 387, I² = 68.5%). The test for subgroup differences was not statistically significant (Q-between = 2.19, df = 1, *p* = .139). The subgroup forest plot is presented in Fig. 4.

These findings do not provide statistical evidence that the magnitude of treatment effects differed by theoretical orientation. However, given the small number of comparisons within each subgroup, this analysis should be interpreted as exploratory. The absence of a statistically significant subgroup difference should not be taken as evidence of equivalence between approaches.

Because interpersonal psychotherapy is the one intervention in the first subgroup whose mechanism is not cognitive or behavioral, we examined whether the comparison depended on its inclusion. Excluding that trial gave g = − 0.66 (95% CI [− 0.78, − 0.54], k = 8) for cognitive–behavioral and interpersonal therapies and g = − 0.50 (95% CI [− 0.71, − 0.30], k = 12) overall, with the test for subgroup differences remaining non-significant (*p* = .064).

### Subgroup analyses by control condition and trial characteristics

Comparisons against inactive control conditions yielded a larger pooled estimate (g = − 0.53, 95% CI [− 0.76, − 0.30], k = 11, *n* = 676, I² = 35.5%) than comparisons against active comparators (g = − 0.22, 95% CI [− 2.19, 1.75], k = 2, *n* = 201, I² = 13.1%), although the difference was not statistically significant (Q-between = 1.76, df = 1, *p* = .184) and the active-comparator stratum contained only two trials. Within the inactive conditions, trials using treatment as usual, usual care or clinical monitoring yielded the largest and most precise estimate (g = − 0.67, 95% CI [− 0.81, − 0.52], k = 5, I² = 0%), whereas waiting-list trials yielded a smaller estimate whose interval included the null (g = − 0.35, 95% CI [− 0.83, 0.13], k = 6, I² = 48.7%); the three-way test was not significant (Q-between = 3.94, df = 2, *p* = .139).

Trials in which anxiety was a designated primary or co-primary outcome yielded a somewhat smaller estimate (g = − 0.32, 95% CI [− 0.80, 0.16], k = 5) than trials in which it was a secondary outcome or in which no outcome hierarchy was stated (g = − 0.56, 95% CI [− 0.81, − 0.31], k = 8), and the difference was not statistically significant (*p* = .25). Only one trial required a numerical cut-off on an anxiety scale at study entry (Moonen et al., 2021; g = − 0.42, 95% CI [− 1.00, 0.15]), so this characteristic could not be examined as a subgroup comparison.

Neither mean age nor the proportion of female participants was associated with effect magnitude in exploratory meta-regressions, whether entered individually (age: b = − 0.046, 95% CI [− 0.154, 0.062], *p* = .37; female: b = − 0.001, 95% CI [− 0.025, 0.022], *p* = .91) or together.

### Sensitivity analysis

To evaluate the robustness of the overall findings, a leave-one-out sensitivity analysis was conducted at trial level. The pooled effect size was recalculated sequentially after removing each trial. Across all thirteen iterations the pooled effect remained statistically significant, ranging from − 0.53 to − 0.43, and no single trial altered the direction of the overall estimate. Residual heterogeneity was stable except in one iteration: omitting Bogosian et al. (2022) reduced I² from 40.7% to 0.0%, whereas every other iteration left it between 34% and 46%. Detailed results are provided in Supplementary Table S2.

A methodological sensitivity analysis was performed by excluding trials rated as having an overall high risk of bias, as prespecified. Exclusion of the six high-risk trials did not attenuate the pooled estimate but increased it (g = − 0.52, 95% CI [− 0.77, − 0.28], k = 7, *n* = 650, I² = 30.2%), and in this restricted analysis the 95% prediction interval excluded the null (− 0.93 to − 0.12). Restriction to the five trials at low risk of bias gave g = − 0.55 (95% CI [− 0.95, − 0.14]). These five trials all evaluated cognitive–behavioral or interpersonal interventions and are therefore not a random subset of the evidence base. Full results are given in Supplementary Table S5.

Restricting the analysis to trials with at least 20 participants per arm yielded a comparable estimate (g = − 0.44, 95% CI [− 0.71, − 0.16], k = 9, *n* = 787), as did exclusion of the four trials for which standard deviations had been derived by conversion or estimated from medians and ranges (g = − 0.50, 95% CI [− 0.80, − 0.21], k = 9, *n* = 548). Substituting the conventional DerSimonian–Laird model produced an identical point estimate with a slightly narrower confidence interval (g = − 0.46, 95% CI [− 0.65, − 0.27]; Supplementary Table S3).

The pooled estimate did not depend on the handling of the three-arm trial. Dividing the shared control group and retaining the two active arms as separate comparisons gave g = − 0.47 (95% CI [− 0.66, − 0.27], 14 comparisons); robust variance estimation with a correlated-effects working model and a small-sample correction gave g = − 0.46 (95% CI [− 0.68, − 0.25]); and a three-level random-effects model gave g = − 0.46 (95% CI [− 0.67, − 0.24]), with the comparison-level variance component estimated at zero. Full results are given in Supplementary Table S7.

### Sensitivity analysis by anxiety measure

Trials using clinician-administered instruments yielded a larger pooled estimate with no detectable heterogeneity (g = − 0.68, 95% CI [− 0.92, − 0.45], k = 4, *n* = 290, I² = 0%) than trials using self-report instruments (g = − 0.35, 95% CI [− 0.63, − 0.06], k = 9, *n* = 587, I² = 44.1%), although the difference was not statistically significant (Q-between = 2.54, df = 1, *p* = .111). Excluding the single trial using the trait subscale of the State–Trait Anxiety Inventory left the estimate essentially unchanged (g = − 0.47, 95% CI [− 0.69, − 0.25], k = 12, *n* = 847).

Because several instruments contain items that overlap with the motor and autonomic features of Parkinson’s disease, measures were also grouped by somatic-item burden. Instruments with substantial somatic content yielded g = − 0.58 (95% CI [− 0.87, − 0.30], k = 7, *n* = 402), whereas instruments with minimal somatic content yielded g = − 0.33 with an interval that included the null (95% CI [− 0.70, 0.05], k = 6, *n* = 475, I² = 50.7%); this difference was likewise not significant (Q-between = 1.64, df = 1, *p* = .200). The heterogeneity within the minimal-somatic stratum reflects the inclusion of Bogosian et al. (2022); the remaining five comparisons in that stratum lie between − 0.24 and − 0.63. All four clinician-administered comparisons evaluated cognitive behavioral interventions and three originate from a single research group, so rating method, research group and intervention type are not separable in these data. These analyses were not prespecified and are exploratory. Full results are given in Supplementary Table S4.

### Publication bias

**Fig. 5:**
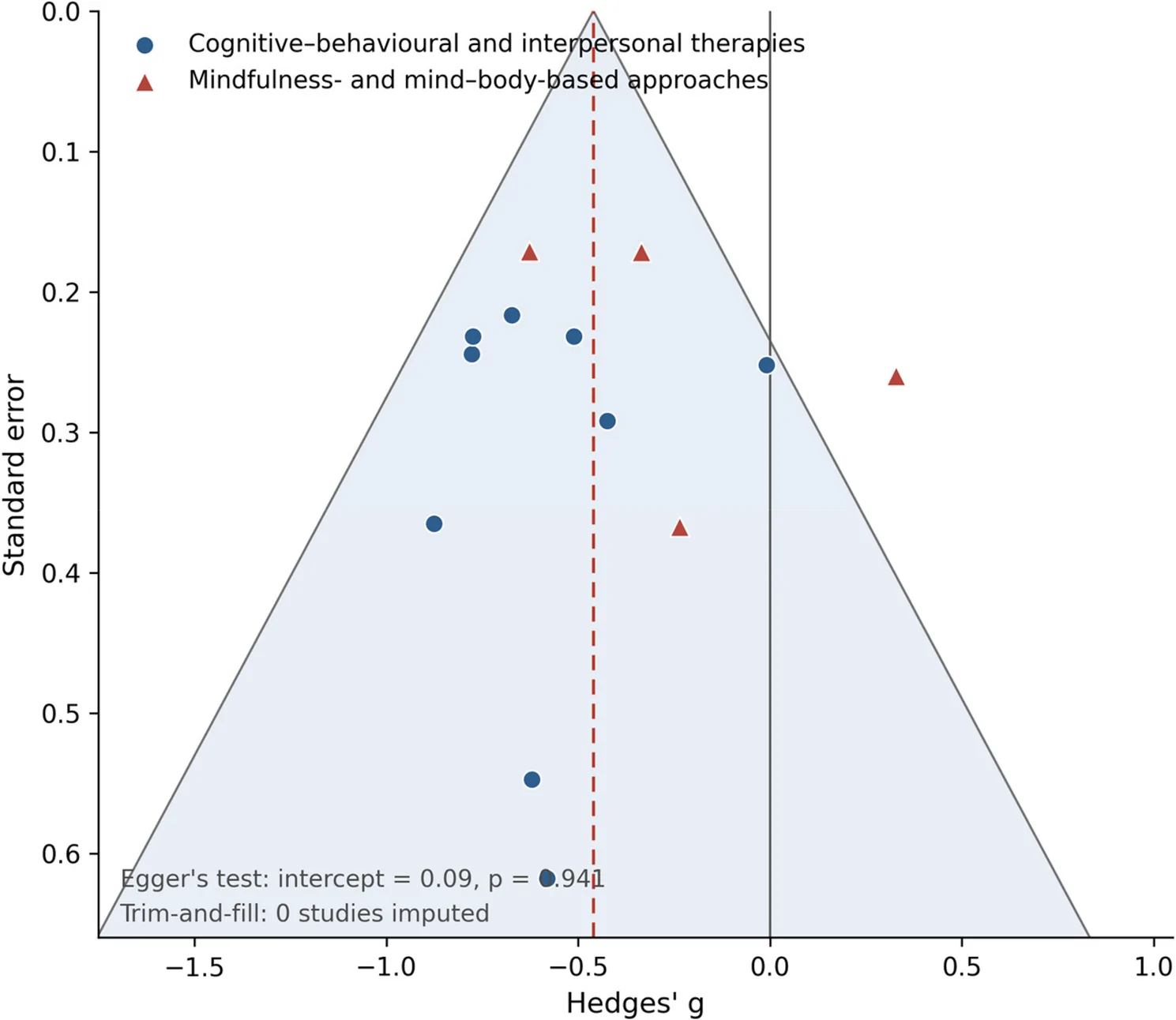
Funnel plot of the thirteen included comparisons, with symbols distinguishing the two intervention families. The dashed vertical line marks the pooled estimate

Visual inspection of the funnel plot did not reveal clear evidence of asymmetry (Fig. 5). Egger’s regression test was non-significant (intercept = 0.09, *p* = .941), as was the rank correlation test (τ = 0.03, *p* = .95), and the trim-and-fill procedure imputed no missing studies.

However, given the relatively small number of included trials, the statistical power to detect small-study effects or publication bias may have been limited. Therefore, while no clear statistical evidence of publication bias was observed, the findings should be interpreted with appropriate caution.

### Certainty of evidence

The certainty of the evidence for the effect of psychological and mind–body interventions on anxiety symptoms was rated very low. The rating was downgraded once for risk of bias, since blinding of participants and providers was not feasible and most anxiety outcomes were self-reported, and once for indirectness, since only one of the thirteen trials required a numerical cut-off on an anxiety scale at entry, anxiety was a designated primary or co-primary outcome in five trials, and eleven of the thirteen comparisons used an inactive control condition. The rating was also downgraded once for inconsistency: between-study heterogeneity was moderate and estimated imprecisely (I² = 40.7%, 95% CI 0% to 79%), the 95% prediction interval included the null (− 1.00 to + 0.08), and one eligible trial estimated an effect in the opposite direction to the remainder. Although sensitivity analysis indicated that the heterogeneity was largely attributable to that trial, the variability could not be regarded as sufficiently explained to avoid downgrading. Imprecision was not downgraded. No empirically established minimally important difference is available for anxiety symptom change in Parkinson’s disease across the instruments used, so a standardized mean difference of 0.20 was adopted as a threshold distinguishing a trivial from at least a small effect; the 95% confidence interval did not cross this threshold, and the total sample of 877 participants exceeded the optimal information size of approximately 800 participants required to detect a standardized mean difference of 0.20 at α = 0.05 with 80% power. The confidence interval did, however, cross the moderate-effect threshold of 0.50, so the precise magnitude of the effect remains uncertain.

Inconsistency was assessed separately for each analysis presented in Table 2. The same judgment was applied to the inactive-control analysis, in which the pattern of variability was comparable (I² = 35.5%; 95% prediction interval − 1.09 to + 0.03), so that this estimate was likewise rated very low. No downgrade for inconsistency was applied to the active-comparator analysis (I² = 13.1%), which was already rated very low on account of serious imprecision, or to the follow-up analysis (I² = 26.0%), which was rated low. The Summary of Findings table is presented as Table 2.

**Table 2:** Summary of Findings: psychological and mind–body interventions for anxiety in Parkinson’s disease. Patient or population: adults with a clinical diagnosis of Parkinson’s disease

Outcome	Effect estimate (Hedges’ g, 95% CI)	№ of participants (studies)	Certainty of the evidence (GRADE)	Comments
Anxiety symptoms at the end of treatment, all comparisons assessed with validated anxiety instruments (HAM-A, HADS-A, BAI, GAI, DASS-A, STAI-T)	−0.46 (− 0.67 to − 0.25)	877 (13 RCTs)	⊕○○○ VERY LOW a, b,c	A standardized reduction of moderate statistical magnitude. The 95% prediction interval was − 1.00 to + 0.08 and therefore included the null, indicating that a future trial in a comparable population could plausibly show no benefit. c
Anxiety symptoms at the end of treatment, comparisons with inactive control conditions (waiting list, treatment as usual, usual care, clinical monitoring)	−0.53 (− 0.76 to − 0.30)	676 (11 RCTs)	⊕○○○ VERY LOW a, b,c	The estimate on which the overall result principally rests. Comparisons of this kind capture therapeutic contact, expectation and attention as well as any effect specific to the intervention. Inconsistency was rated down for this analysis on the same basis as for the overall estimate (I² = 35.5%; 95% prediction interval − 1.09 to + 0.03).
Anxiety symptoms at the end of treatment, comparisons with active comparators (supportive therapy, stretching and resistance exercise)	−0.22 (− 2.19 to + 1.75)	201 (2 RCTs)	⊕○○○ VERY LOW a, b,e	Downgraded additionally for serious imprecision: only two trials contribute, and the interval is compatible with a substantial benefit, no effect, or harm. The evidence bearing on intervention-specific effects is correspondingly weak.
Anxiety symptoms at follow-up (8 weeks to 6 months after the end of the intervention)	−0.39 (− 0.60 to − 0.18)	708 (9 RCTs)	⊕⊕○○ LOW a, b,d	A similar pooled reduction was observed at follow-up. Inconsistency was not rated down for this outcome (I² = 26.0%). However, follow-up intervals varied, and monthly booster sessions were permitted in two trials; durable maintenance after complete treatment cessation was not established.

Patient or population: adults with a clinical diagnosis of Parkinson’s disease

Setting: outpatient, community and remotely delivered settings in eight countries and one special administrative region

Intervention: structured psychological or mind–body intervention (cognitive–behavioral and interpersonal therapies; mindfulness- and mind–body-based approaches)

Comparison: waiting list, treatment as usual, usual care, clinical monitoring, or an active comparator

## Discussion

This meta-analysis found that psychological and mind–body interventions were associated with a statistically significant reduction in anxiety symptoms among individuals with Parkinson’s disease. The pooled effect size corresponded to a standardized reduction of moderate statistical magnitude (Hedges’ g = − 0.46), and twelve of the thirteen comparisons favored the intervention. Because the included trials used different anxiety measures, including the HAM-A, HADS-A, BAI, DASS-A, GAI and STAI-T, the pooled estimate was expressed as a standardized mean difference rather than as a raw mean difference. Although this approach allowed results across different scales to be synthesized on a common metric, standardized mean differences are less directly interpretable in clinical terms than scale-specific raw-score changes. The observed effect should therefore be interpreted as a standardized reduction of moderate statistical magnitude rather than as a precise estimate of change on any single anxiety measure. A conventional classification of a standardized effect does not in itself establish that the improvement is clinically meaningful, particularly when different anxiety instruments and clinically heterogeneous populations have been combined. Given the close association between anxiety and diminished quality of life, sleep disturbance and functional impairment in PD, reductions in anxiety symptoms may nevertheless be relevant to patient functioning, although the clinical importance of the pooled standardized effect cannot be established from the present data.

Two features of the evidence qualify this conclusion. The 95% prediction interval extended from − 1.00 to + 0.08 and therefore included the null: although the average effect across the existing trials is a reduction in anxiety, the data are compatible with a future trial in a comparable population showing no benefit. In addition, six of the thirteen trials were judged to be at high risk of bias overall and two raised some concerns, principally because blinding of participants and providers is not feasible for psychological interventions and most anxiety outcomes were self-reported. The overall certainty of the evidence was accordingly rated very low.

In clinical contexts where pharmacological management is complicated by adverse effects and polypharmacy burden, psychological interventions may warrant consideration as complementary approaches, although the very low certainty of the evidence precludes firm treatment recommendations.

Between-study heterogeneity was moderate (I² = 40.7%, 95% CI 0% to 79%; τ² = 0.052, 95% CI 0.000 to 0.238). Given the small number of included comparisons, these estimates are imprecise, and the confidence intervals remain compatible with both homogeneity and substantial variation. The heterogeneity estimate was nonetheless strongly influenced by a single trial, although uncertainty around it remained substantial. Omitting Bogosian et al. (2022) reduced I² from 40.7% to 0%, whereas omitting any other single trial left it between 34% and 46%. That trial was the only one of the thirteen in which anxiety scores were higher in the intervention arm at post-treatment, and three features of its design bear on this: it was designed and reported as a feasibility rather than an efficacy trial; its investigators deliberately chose not to use anxiety or depressive symptoms as entry criteria; and mean baseline anxiety was accordingly close to the instrument’s clinical threshold in both arms. The trial met every prespecified eligibility criterion and was retained in the primary analysis, since removing a trial because it introduces heterogeneity would substitute a post hoc statistical criterion for a prespecified clinical one.

Subgroup analyses showed a significant reduction in anxiety for cognitive–behavioral and interpersonal therapies (g = − 0.57, I² = 0%), whereas the estimate for mindfulness- and mind–body-based approaches was smaller and its confidence interval included the null (g = − 0.25, I² = 68.5%); the test for subgroup differences was not statistically significant. This should not be read as evidence that one family of interventions is superior to the other. The mindfulness subgroup contains only four comparisons, one of which is the feasibility trial discussed above, and none of the four was rated at low risk of bias, whereas five of the nine cognitive–behavioral and interpersonal trials were; intervention type, risk of bias and outcome instrument are therefore not separable in these data. The present meta-analysis synthesized symptom-level anxiety outcomes only and did not include any mechanistic or mediational data, so the processes described in the remainder of this paragraph are offered as interpretive hypotheses rather than as processes established here. Cognitive–behavioral and interpersonal therapies may operate through modification of maladaptive cognitions, enhancement of coping efficacy and change in interpersonal patterns, thereby strengthening emotion regulation. Mindfulness- and mind–body-based approaches may instead reduce experiential avoidance and promote nonjudgmental awareness of bodily sensations, thereby attenuating emotional reactivity and physiological arousal. Shared processes such as improved emotion regulation, enhanced attentional control and altered appraisal of somatic experiences may be particularly relevant in PD, where motor symptoms and autonomic arousal interact with anxiety.

A similar reduction was observed at follow-up. Among the nine trials contributing a follow-up comparison in which the control group had not crossed over, the pooled estimate was g = − 0.39 (95% CI − 0.60 to − 0.18), against g = − 0.41 for the same nine trials at the end of treatment. This comparison is provisional. The two estimates were not compared statistically; the follow-up intervals were not standardized, ranging from 8 weeks to 6 months; and the nine trials are not a random subset of the evidence base, since a waiting-list design cannot in principle sustain an untreated comparison beyond the point at which the waiting period ends. In two trials monthly booster sessions were permitted in the intervention arm during an extension phase, so these estimates reflect effects observed at post-treatment follow-up or during an extension phase rather than maintenance after complete treatment cessation. A similar pooled reduction was observed at follow-up, but the analysis does not establish durable maintenance after complete treatment cessation.

The pooled effect against inactive control conditions (11 comparisons, g = − 0.53, 95% CI − 0.76 to − 0.30) was numerically larger than that against active comparators (2 comparisons, g = − 0.22, 95% CI − 2.19 to + 1.75), although the difference was not statistically significant and the active-comparator stratum contains only two trials. This asymmetry is the most important constraint on what the present findings can support. Eleven of thirteen comparisons were made against waiting lists, treatment as usual, usual care or clinical monitoring, and comparisons of this kind capture not only the specific effects of the intervention but also therapeutic contact, expectation and attention. The pooled estimate should therefore be understood as the effect of adding a structured psychological intervention relative to a waiting list, usual care, treatment as usual, or clinical monitoring, rather than as an estimate of benefit attributable to a specific therapeutic mechanism. The two active comparators were supportive therapy and a structured stretching and resistance exercise program; neither is an attention placebo, and the absence of a demonstrated difference should not be read as an absence of efficacy.

Six different anxiety instruments were used across the thirteen trials, and the pooled estimate was not uniform across them. Trials using clinician-administered instruments yielded a larger effect with no detectable heterogeneity (g = − 0.68, I² = 0%) than trials using self-report instruments (g = − 0.35, I² = 44.1%), a direction consistent with detection bias where outcome assessors could not be blinded, although the difference was not statistically significant. Because several instruments contain items overlapping with the motor and autonomic features of PD, measures were also grouped by somatic-item burden. Instruments with substantial somatic content yielded g = − 0.58, whereas instruments with minimal somatic content yielded g = − 0.33 with an interval including the null; the heterogeneity in the latter stratum reflects the inclusion of the feasibility trial identified above, and the remaining five comparisons lie between − 0.24 and − 0.63. The instruments also differ in what they measure. The STAI-T assesses stable trait anxiety and may be less responsive to short-term treatment, whereas the HADS-A and GAI were developed to minimize somatic content in medically ill and older populations. The possibility that part of the observed effect reflects change in somatic items rather than in anxiety itself cannot be excluded on the present data.

The pooled estimate was not attenuated after trials at high risk of bias were excluded, although this sensitivity analysis does not eliminate concerns regarding methodological bias. Excluding the six trials at high risk of bias did not attenuate the pooled estimate but increased it (g = − 0.52, 95% CI − 0.77 to − 0.28), and in this restricted analysis the prediction interval excluded the null. Restricting further to the five trials at low risk of bias gave g = − 0.55. These five trials all evaluated cognitive–behavioral or interpersonal interventions and are therefore not a random subset of the evidence base.

By synthesizing evidence from thirteen randomized controlled trials, the present meta-analysis provides a quantitative estimate of treatment efficacy, and the pooled estimate remained statistically significant in every leave-one-out iteration, ranging from − 0.53 to − 0.43. Our estimate is substantially smaller than that reported by the most recent overlapping review, which gave a pooled standardized mean difference of − 2.00 for the anxiety outcome [13]. Effects of that magnitude correspond to differences of two or more pooled standard deviations and are rarely observed in the psychotherapy literature. The discrepancy may be attributable, at least in part, to differences in effect-size derivation: that review computed effect sizes from change-from-baseline scores with imputed dispersion values, whereas the present review used post-treatment values. Differences in the included evidence base, including trials retrieved from Chinese-language databases, and differences in search coverage may also have contributed.

This study extends prior literature by directly comparing cognitive–behavioral and interpersonal therapies with mindfulness- and mind–body-based approaches within a unified analytic framework. The absence of statistically significant subgroup differences may suggest the possibility of shared therapeutic processes across intervention models, but this interpretation remains tentative given the limited number of trials in each subgroup and the confounding described above.

The present findings indicate that psychological interventions are associated with a standardized reduction of moderate statistical magnitude in anxiety symptoms in individuals with PD. This reinforces evidence supporting cognitive behavioral therapy and suggests that diverse delivery modalities and therapeutic frameworks may be associated with reductions in anxiety symptoms in this population. Anxiety in PD is often intertwined with realistic and persistent stressors, including motor fluctuations, progressive functional decline and fall risk. Within this context, interventions that aim solely to reduce anxiety symptoms may be insufficient, and approaches that facilitate adaptive functioning despite the presence of anxiety may hold particular value. This proposed pathway is not tested by the present data, which comprise anxiety symptom scores alone; it is presented here as a hypothesis warranting future longitudinal and mediational investigation.

Mindfulness- and mind–body-based interventions emphasize present-moment awareness and acceptance-oriented coping rather than symptom elimination [38]. The observation that such approaches were also associated with reductions in anxiety, albeit with an interval including the null, supports the potential clinical relevance of strategies that foster nonjudgmental awareness of bodily sensations and sustained engagement in valued activities, although direct evidence for equivalence with cognitive–behavioral and interpersonal therapies remains insufficient. Integrative treatment models addressing both cognitive restructuring and experiential, body-based processes may be especially beneficial for PD-related anxiety, particularly when targeting fear of falling and activity avoidance; this too is a hypothesis for future research rather than a conclusion supported by the present synthesis.

Although heterogeneity was moderate and was strongly influenced by a single trial, the findings suggest that theoretically informed psychological treatments may have potential across varied clinical contexts, although the very low certainty of the evidence precludes firm clinical conclusions. The present analysis does not support the superiority of one model over the other.

A methodological strength of this review is that the quantitative synthesis was restricted to randomized controlled trials, which supports a more internally valid estimate of intervention effects on anxiety outcomes. Anxiety was explicitly defined as the primary outcome of the review, and interventions were categorized according to theoretical orientation, thereby reflecting diversity in therapeutic mechanisms that has not been sufficiently examined in prior meta-analyses. Further strengths are a search conducted to a single common date across four databases, duplicate independent assessment at full text and for risk of bias, the use of restricted maximum likelihood estimation with the Hartung–Knapp adjustment for all pooled estimates, and the reporting of a prediction interval alongside the confidence interval.

Several limitations warrant consideration. First, the certainty of the evidence is limited by the small number of included trials and by the fact that six were rated as having a high risk of bias; the stability of the estimate across sensitivity analyses should not be interpreted as eliminating concerns regarding methodological bias. With thirteen comparisons the between-study variance cannot be estimated with useful precision, the prediction interval includes the null, and clinical and methodological diversity is not captured by I² at all. Second, the review combines trials that address related but distinct clinical questions: only one trial required a numerical cut-off on an anxiety scale at entry and anxiety was a secondary outcome in seven, so the evidence speaks less directly to the treatment of clinically significant anxiety than the pooled estimate alone might suggest. Third, only two comparisons used an active comparator, the corresponding estimate is imprecise, and both comparators were themselves potentially efficacious interventions; the contribution of non-specific factors therefore cannot be separated from specific treatment effects. Fourth, title and abstract screening was conducted by a single reviewer, eligibility was restricted to reports published in English, two trials retrievable only through Chinese-language databases were not included, PsycINFO could not be searched, and standard deviations were derived by conversion in three trials and estimated from medians and ranges in one.

Three priorities for future research follow from these limitations. Trials should recruit participants with clinically significant anxiety rather than treating anxiety as a secondary outcome in samples selected on other grounds. Active comparators are needed, since only two of thirteen comparisons currently permit any separation of specific from non-specific effects. And instruments with minimal somatic content should be preferred, or reported alongside conventional measures, given that the pooled effect was smallest and least precise in exactly those instruments. Large-scale trials with extended and standardized follow-up are needed to clarify the durability of treatment effects and to elucidate the mechanisms of change.

## Conclusions

This systematic review and meta-analysis suggests that psychological and mind–body interventions may reduce anxiety symptoms among individuals with Parkinson’s disease, with a pooled standardized effect of Hedges’ g = − 0.46, and a similar reduction was observed at follow-up. Cognitive–behavioral and interpersonal therapies showed a significant reduction with no detectable heterogeneity, whereas the estimate for mindfulness- and mind–body-based approaches was smaller and imprecise; no statistically significant subgroup difference was observed. This subgroup analysis was exploratory and should not be interpreted as evidence of equivalence between approaches.

The estimate was robust to the exclusion of trials at high risk of bias and to the method used to handle the one multiarm trial. It rests, however, on a small evidence base, on comparisons made predominantly against inactive control conditions, and on a prediction interval that admits the possibility of no benefit in a future trial, and the overall certainty of the evidence was rated very low. Anxiety in PD has been linked to fear of falling, activity avoidance, functional decline and reduced quality of life, which raises the possibility that interventions may need to extend beyond symptom reduction to address avoidance behaviors and promote sustained engagement in meaningful activities. Cognitive restructuring strategies and approaches emphasizing experiential acceptance and behavioral persistence may both be clinically relevant, but their specific mechanisms of action were not evaluated in the present meta-analysis and require direct investigation in future trials.

## Supplementary Information

Supplementary Material 1.

## Data Availability

All data analysed during this study are included in this published article and its supplementary information files. The data were extracted from previously published, publicly available randomized controlled trials, which are cited in the reference list.
