# Supplementary material for: The efficacy of psychological interventions for anxiety in Parkinson’s disease: a systematic review and meta-analysis including mindfulness and mind–body approaches
Source: BMC Psychol. 2026 Aug 26;14:1512. doi: 10.1186/s40359-026-05436-y (PMC13685121; doi:10.1186/s40359-026-05436-y)
Supplement: Supplementary file 1 [file 40359_2026_5436_MOESM1_ESM.docx]

**Supplementary Materials**

**Supplementary Appendix A Search strategies**

Searches were conducted by a medical librarian. The initial search of PubMed, Embase (via Elsevier) and the Cochrane Central Register of Controlled Trials was run on 10 February 2026. Following peer review, Web of Science was added and the search was re-run across all four databases on 27 July 2026, so that a single search date applies to every source. The search strategies for PubMed, Embase and the Cochrane Library were identical on the two dates; only the numbers of records retrieved differ, and both are given below. No date, language or publication-type limits were applied within the databases; the restriction to reports published in English was applied at the screening stage. The reference lists of the included trials and of previous reviews were additionally screened.

**Table A1 Records retrieved by database and search date**

| **Database** | **Interface** | **Records retrieved 10 February 2026** | **Records retrieved 27 July 2026** |
| --- | --- | --- | --- |
| PubMed | NLM | 5,095 | 5,290 |
| Embase | Elsevier | 9,003 | 9,390 |
| Cochrane Central Register of Controlled Trials | Wiley | 1,389 | 1,474 |
| Web of Science | Clarivate | not searched | 4,184 |
| **Total** |  | **15,487** | **20,338** |
| Records removed as duplicates within each search (EndNote) |  | 4,327 | 7,334 |
| **Records remaining after within-search deduplication** |  | **11,160** | **13,004** |

Records from the two searches were subsequently combined and deduplicated against one another in EndNote, so that records common to the two searches were counted once. Of the 35,825 records retrieved in total, 23,211 were removed as duplicate or overlapping records, leaving 12,614 records for title and abstract screening (Figure 1).

**Table A2 PubMed search strategy**

| **#** | **Search query** | **Records 10 Feb 2026** | **Records 27 Jul 2026** |
| --- | --- | --- | --- |
| #1 | "Parkinson Disease"[Mesh] | 93545 | 95758 |
| #2 | "Parkinson Disease"[TW] OR "Idiopathic Parkinson Disease"[TW] OR "Idiopathic Parkinson's Disease"[TW] OR "Lewy Body Parkinson Disease"[TW] OR "Lewy Body Parkinson's Disease"[TW] OR "Paralysis Agitans"[TW] OR "Parkinson Disease, Idiopathic"[TW] OR "Parkinson's Disease"[TW] OR "Parkinson's Disease, Idiopathic"[TW] OR "Parkinson's Disease, Lewy Body"[TW] OR "Primary Parkinsonism"[TW] OR "Parkinsonism, Primary"[TW] OR "idiopathic parkinsonism"[TW] OR "Lewy bodies of Parkinson disease"[TW] OR "Lewy bodies of Parkinson's disease"[TW] OR "Lewy bodies of Parkinsons disease"[TW] OR "Lewy body Parkinsons disease"[TW] OR "Parkinson dementia complex"[TW] OR "Parkinsons disease"[TW] | 158073 | 163040 |
| #3 | #1 OR #2 | 158073 | 163040 |
| #4 | "Psychotherapy"[Mesh] | 237174 | 241814 |
| #5 | "Psychotherapy"[TW] OR "Psychotherapies"[TW] OR "holistic psychotherapy"[TW] OR "multiple psychotherapy"[TW] OR "psychotherapeutic processes"[TW] OR "psychotherapeutic training"[TW] OR "psychotherapy, multiple"[TW] OR "socioenvironmental therapy"[TW] | 102434 | 104069 |
| #6 | "Psychology, Clinical"[Mesh] | 3361 | 3376 |
| #7 | "Psychology, Clinical"[TW] OR "Clinical Psychology"[TW] OR "clinical psychologist"[TW] OR "psychologist, clinical"[TW] | 6507 | 6556 |
| #8 | "Acceptance and Commitment Therapy"[Mesh] | 1329 | 1406 |
| #9 | "Acceptance and Commitment Therapy"[TW] OR "acceptance commitment therapy"[TW] OR "ACT psychotherapy"[TW] OR "ACT"[TW] OR "Psychological flexibility"[TW] OR "Acceptance-based"[TW] OR "Cognitive defusion"[TW] OR "Values-based"[TW] OR "Committed action"[TW] | 384962 | 395075 |
| #10 | ("Cognitive Behavioral Therapy"[Mesh] AND "Mindfulness"[Mesh]) OR "Mindfulness-Based Cognitive Therapy"[Mesh] | 8668 | 9170 |
| #11 | "Mindfulness-Based Cognitive Therapy"[TW] OR "Cognitive Therapy, Mindfulness-Based"[TW] OR "Mindfulness-Based Cognitive Therapies"[TW] OR "Mindfulness Based Cognitive Therapy"[TW] OR "Therapy, Mindfulness-Based Cognitive"[TW] OR "MBCT"[TW] OR "mindfulness cognitive therapy"[TW] OR "mindfulness-based CBT"[TW] OR "mindfulness-based cognitive behavior therapy"[TW] OR "mindfulness-based cognitive behavioral therapy"[TW] OR "mindfulness-based cognitive behavior therapy"[TW] OR "mindfulness-based cognitive behavioral therapy"[TW] OR "mindfulness-CBT"[TW] | 1183 | 1240 |
| #12 | "Cognitive Behavioral Therapy"[TW] OR "Behavioral Therapies, Cognitive"[TW] OR "Behavioral Therapy, Cognitive"[TW] OR "Cognitive Behavioral Therapies"[TW] OR "Therapies, Cognitive Behavioral"[TW] OR "Therapy, Cognitive Behavioral"[TW] OR "Cognition Therapy"[TW] OR "Cognition Therapies"[TW] OR "Therapies, Cognition"[TW] OR "Therapy, Cognitive Behavior"[TW] OR "Behavior Therapies, Cognitive"[TW] OR "Cognitive Behavior Therapies"[TW] OR "Therapies, Cognitive Behavior"[TW] OR "Therapy, Cognition"[TW] OR "Behavior Therapy, Cognitive"[TW] OR "Cognitive Behavior Therapy"[TW] OR "Cognitive Psychotherapy"[TW] OR "Cognitive Psychotherapies"[TW] OR "Psychotherapies, Cognitive"[TW] OR "Psychotherapy, Cognitive"[TW] OR "Therapy, Cognitive"[TW] OR "Cognitive Therapies"[TW] OR "Therapies, Cognitive"[TW] OR "Cognitive Behavior Therapy"[TW] OR "Behavior Therapies, Cognitive"[TW] OR "Behavior Therapy, Cognitive"[TW] OR "Cognitive Behavior Therapies"[TW] OR "Therapies, Cognitive Behavior"[TW] OR "Therapy, Cognitive Behavior"[TW] OR "Cognitive Therapy"[TW] OR "CBT"[TW] OR "cognitive behavior intervention"[TW] OR "cognitive behavior treatment"[TW] OR "cognitive behavioral intervention"[TW] OR "cognitive behavioral treatment"[TW] OR "cognitive behavior intervention"[TW] OR "cognitive behavior treatment"[TW] OR "cognitive behavioral intervention"[TW] OR "cognitive behavioral therapy"[TW] OR "cognitive behavioral treatment"[TW] OR "Third-wave CBT"[TW] | 53706 | 55509 |
| #13 | "Mindfulness"[TW] OR "Mindfulness Meditation"[TW] OR "Meditation, Mindfulness"[TW] OR "Mindfulness Meditations"[TW] | 19072 | 20300 |
| #14 | ("Mindfulness"[Mesh] AND "Stress, Psychological"[Mesh]) OR "Mindfulness-Based Stress Reduction"[Mesh] | 2378 | 2529 |
| #15 | "Mindfulness-Based Stress Reduction"[TW] OR "Mindfulness Based Stress Reduction"[TW] OR "Mindfulness-Based Stress Reductions"[TW] OR "Stress Reduction, Mindfulness-Based"[TW] OR "MBSR Therapy"[TW] OR "MBSR Therapies"[TW] OR "Therapy, MBSR"[TW] OR "Mindfulness-Based Stress Reduction Therapy"[TW] OR "Mindfulness Based Stress Reduction Therapy"[TW] OR "MBSR"[TW] OR "mindfulness stress reduction course"[TW] OR "mindfulness stress reduction program"[TW] OR "mindfulness stress reduction training"[TW] OR "mindfulness stress reduction training program"[TW] OR "mindfulness-based stress reduction technique"[TW] OR "wellness-based mindfulness stress reduction intervention"[TW] OR "Mindfulness-Based"[TW] | 6857 | 7347 |
| #16 | "Meditation"[Mesh] | 4356 | 4460 |
| #17 | "Meditation"[TW] OR "Transcendental Meditation"[TW] OR "Meditation, Transcendental"[TW] | 9886 | 10236 |
| #18 | "Yoga"[Mesh] | 4538 | 4720 |
| #19 | "Yoga"[TW] OR "yogic meditation"[TW] | 9251 | 9737 |
| #20 | "Muscle Stretching Exercises"[Mesh] | 2301 | 2365 |
| #21 | "Muscle Stretching Exercises"[TW] OR "Exercise, Muscle Stretching"[TW] OR "Muscle Stretching Exercise"[TW] OR "Active Stretching"[TW] OR "Stretching, Active"[TW] OR "Static-Active Stretching"[TW] OR "Static Active Stretching"[TW] OR "Stretching, Static-Active"[TW] OR "Proprioceptive Neuromuscular"[TW] OR "Facilitation (PNF) Stretching"[TW] OR "Proprioceptive Neuromuscular Facilitation"[TW] OR "Neuromuscular Facilitation, Proprioceptive"[TW] OR "Proprioceptive Neuromuscular Facilitations"[TW] OR "PNF Stretching"[TW] OR "PNF Stretchings"[TW] OR "Stretching, PNF"[TW] OR "PNF Stretching Exercise"[TW] OR "Exercise, PNF Stretching"[TW] OR "PNF Stretching Exercises"[TW] OR "Stretching Exercise, PNF"[TW] OR "Passive Stretching"[TW] OR "Stretching, Passive"[TW] OR "Static-Passive Stretching"[TW] OR "Static Passive Stretching"[TW] OR "Stretching, Static-Passive"[TW] OR "Relaxed Stretching"[TW] OR "Stretching, Relaxed"[TW] OR "Static Stretching"[TW] OR "Stretching, Static"[TW] OR "Isometric Stretching"[TW] OR "Stretching, Isometric"[TW] OR "Dynamic Stretching"[TW] OR "Stretching, Dynamic"[TW] OR "Ballistic Stretching"[TW] OR "Stretching, Ballistic"[TW] OR "stretching exercises"[TW] OR "stretching exercise"[TW] | 4962 | 5130 |
| #22 | "Tai Ji"[Mesh] | 1758 | 1826 |
| #23 | "Tai Ji"[TW] OR "Tai-ji"[TW] OR "Tai Chi"[TW] OR "Chi, Tai"[TW] OR "Tai Chi Chuan"[TW] OR "Taiji"[TW] OR "Taijiquan"[TW] OR "T'ai Chi"[TW] OR "Tai Ji Quan"[TW] OR "Ji Quan, Tai"[TW] OR "Quan, Tai Ji"[TW] OR "Taiji quan"[TW] | 3404 | 3616 |
| #24 | "Qigong"[Mesh] | 465 | 482 |
| #25 | "Qigong"[TW] OR "Ch'i Kung"[TW] OR "Qi Gong"[TW] OR "chi kung"[TW] OR "chigung"[TW] | 1523 | 1599 |
| #26 | "Dance Therapy"[Mesh] | 554 | 578 |
| #27 | "Dance Therapy"[TW] OR "Therapy, Dance"[TW] OR "Dance Therapies"[TW] OR "Therapies, Dance"[TW] OR "dance movement psychotherapy"[TW] OR "dance movement therapy"[TW] OR "dance psychotherapy"[TW] | 805 | 851 |
| #28 | "Exercise Therapy"[Mesh] | 74259 | 76719 |
| #29 | "Exercise Therapy"[TW] OR "Exercise Prescription"[TW] OR "Rehabilitation Exercise"[TW] OR "Exercise, Rehabilitation"[TW] OR "Exercises, Rehabilitation"[TW] OR "Rehabilitation Exercises"[TW] OR "Remedial Exercise"[TW] OR "Exercise, Remedial"[TW] OR "Exercises, Remedial"[TW] OR "Remedial Exercises"[TW] OR "Therapy, Exercise"[TW] OR "Exercise Therapies"[TW] OR "Therapies, Exercise"[TW] OR "Physical Activity Intervention"[TW] OR "motion therapy"[TW] OR "movement-based therapy"[TW] OR "movement therapy"[TW] OR "Movement Therapies"[TW] OR "motion therapies"[TW] | 69410 | 71885 |
| #30 | "Mind-Body Therapies"[Mesh] | 51465 | 52420 |
| #31 | "Mind-Body Therapies"[TW] OR "Mind Body Therapies"[TW] OR "Mind-Body Therapy"[TW] OR "Therapies, Mind-Body"[TW] OR "Therapy, Mind-Body"[TW] OR "Mind-Body Medicine"[TW] OR "Mind Body Medicine"[TW] OR "alternative therapies"[TW] OR "alternative therapy"[TW] OR "complementary medicine"[TW] OR "complementary therapies"[TW] OR "eclectic medicine"[TW] OR "eclecticism, historical"[TW] OR "historical eclecticism"[TW] OR "mental healing"[TW] OR "metaphysical mind-body relations"[TW] OR "mind body technique"[TW] OR "mind body therapy"[TW] OR "mind-body relations (metaphysics)"[TW] OR "mind-body relations, metaphysical"[TW] OR "polarity therapy"[TW] OR "radiaesthesia"[TW] OR "radiesthesia"[TW] OR "therapeutic cults"[TW] OR "Thomsonian medicine"[TW] OR "alternative medicine"[TW] OR "Mind-body exercise"[TW] | 48032 | 49120 |
| #32 | #4 OR #5 OR #6 OR #7 OR #8 OR #9 OR #10 OR #11 OR #12 OR #13 OR #14 OR #15 OR #16 OR #17 OR #18 OR #19 OR #20 OR #21 OR #22 OR #23 OR #24 OR #25 OR #26 OR #27 OR #28 OR #29 OR #30 OR #31 | 800258 | 821450 |
| #33 | #3 AND #32 | 5095 | 5290 |

**Table A3 Embase (Elsevier) search strategy**

| **#** | **Search query** | **Records 10 Feb 2026** | **Records 27 Jul 2026** |
| --- | --- | --- | --- |
| #1 | "Parkinson disease"/exp | 238685 | 246630 |
| #2 | "Parkinson Disease":ti,ab,kw OR "Idiopathic Parkinson Disease":ti,ab,kw OR "Idiopathic Parkinson^s Disease":ti,ab,kw OR "Lewy Body Parkinson Disease":ti,ab,kw OR "Lewy Body Parkinson^s Disease":ti,ab,kw OR "Paralysis Agitans":ti,ab,kw OR "Parkinson Disease, Idiopathic":ti,ab,kw OR "Parkinson^s Disease":ti,ab,kw OR "Parkinson^s Disease, Idiopathic":ti,ab,kw OR "Parkinson^s Disease, Lewy Body":ti,ab,kw OR "Primary Parkinsonism":ti,ab,kw OR "Parkinsonism, Primary":ti,ab,kw OR "idiopathic parkinsonism":ti,ab,kw OR "Lewy bodies of Parkinson disease":ti,ab,kw OR "Lewy bodies of Parkinson^s disease":ti,ab,kw OR "Lewy bodies of Parkinsons disease":ti,ab,kw OR "Lewy body Parkinsons disease":ti,ab,kw OR "Parkinson dementia complex":ti,ab,kw OR "Parkinsons disease":ti,ab,kw | 214231 | 221247 |
| #3 | #1 OR #2 | 269413 | 277871 |
| #4 | "psychotherapy"/exp | 381550 | 393472 |
| #5 | "Psychotherapy":ti,ab,kw OR "Psychotherapies":ti,ab,kw OR "holistic psychotherapy":ti,ab,kw OR "multiple psychotherapy":ti,ab,kw OR "psychotherapeutic processes":ti,ab,kw OR "psychotherapeutic training":ti,ab,kw OR "psychotherapy, multiple":ti,ab,kw OR "socioenvironmental therapy":ti,ab,kw | 74237 | 76123 |
| #6 | "clinical psychology"/exp | 8637 | 8821 |
| #7 | "Psychology, Clinical":ti,ab,kw OR "Clinical Psychology":ti,ab,kw OR "clinical psychologist":ti,ab,kw OR "psychologist, clinical":ti,ab,kw | 6750 | 6994 |
| #8 | "acceptance and commitment therapy"/exp | 4501 | 4907 |
| #9 | "Acceptance and Commitment Therapy":ti,ab,kw OR "acceptance commitment therapy":ti,ab,kw OR "ACT psychotherapy":ti,ab,kw OR "ACT":ti,ab,kw OR "Psychological flexibility":ti,ab,kw OR "Acceptance-based":ti,ab,kw OR "Cognitive defusion":ti,ab,kw OR "Values-based":ti,ab,kw OR "Committed action":ti,ab,kw | 469946 | 483062 |
| #10 | "mindfulness-based cognitive therapy"/exp | 1149 | 1272 |
| #11 | "Mindfulness-Based Cognitive Therapy":ti,ab,kw OR "Cognitive Therapy, Mindfulness-Based":ti,ab,kw OR "Mindfulness-Based Cognitive Therapies":ti,ab,kw OR "Mindfulness Based Cognitive Therapy":ti,ab,kw OR "Therapy, Mindfulness-Based Cognitive":ti,ab,kw OR "MBCT":ti,ab,kw OR "mindfulness cognitive therapy":ti,ab,kw OR "mindfulness-based CBT":ti,ab,kw OR "mindfulness-based cognitive behavior therapy":ti,ab,kw OR "mindfulness-based cognitive behavioral therapy":ti,ab,kw OR "mindfulness-based cognitive behavior therapy":ti,ab,kw OR "mindfulness-based cognitive behavioral therapy":ti,ab,kw OR "mindfulness-CBT":ti,ab,kw | 1867 | 1964 |
| #12 | "cognitive behavioral therapy"/exp | 48325 | 51950 |
| #13 | "Cognitive Behavioral Therapy":ti,ab,kw OR "Behavioral Therapies, Cognitive":ti,ab,kw OR "Behavioral Therapy, Cognitive":ti,ab,kw OR "Cognitive Behavioral Therapies":ti,ab,kw OR "Therapies, Cognitive Behavioral":ti,ab,kw OR "Therapy, Cognitive Behavioral":ti,ab,kw OR "Cognition Therapy":ti,ab,kw OR "Cognition Therapies":ti,ab,kw OR "Therapies, Cognition":ti,ab,kw OR "Therapy, Cognitive Behavior":ti,ab,kw OR "Behavior Therapies, Cognitive":ti,ab,kw OR "Cognitive Behavior Therapies":ti,ab,kw OR "Therapies, Cognitive Behavior":ti,ab,kw OR "Therapy, Cognition":ti,ab,kw OR "Behavior Therapy, Cognitive":ti,ab,kw OR "Cognitive Behavior Therapy":ti,ab,kw OR "Cognitive Psychotherapy":ti,ab,kw OR "Cognitive Psychotherapies":ti,ab,kw OR "Psychotherapies, Cognitive":ti,ab,kw OR "Psychotherapy, Cognitive":ti,ab,kw OR "Therapy, Cognitive":ti,ab,kw OR "Cognitive Therapies":ti,ab,kw OR "Therapies, Cognitive":ti,ab,kw OR "Cognitive Behavior Therapy":ti,ab,kw OR "Behavior Therapies, Cognitive":ti,ab,kw OR "Behavior Therapy, Cognitive":ti,ab,kw OR "Cognitive Behavior Therapies":ti,ab,kw OR "Therapies, Cognitive Behavior":ti,ab,kw OR "Therapy, Cognitive Behavior":ti,ab,kw OR "Cognitive Therapy":ti,ab,kw OR "CBT":ti,ab,kw OR "cognitive behavior intervention":ti,ab,kw OR "cognitive behavior treatment":ti,ab,kw OR "cognitive behavioral intervention":ti,ab,kw OR "cognitive behavioral treatment":ti,ab,kw OR "cognitive behavior intervention":ti,ab,kw OR "cognitive behavior treatment":ti,ab,kw OR "cognitive behavioral intervention":ti,ab,kw OR "cognitive behavioral therapy":ti,ab,kw OR "cognitive behavioral treatment":ti,ab,kw OR "Third-wave CBT":ti,ab,kw | 62975 | 65559 |
| #14 | "mindfulness"/exp | 26674 | 28681 |
| #15 | "Mindfulness":ti,ab,kw OR "Mindfulness Meditation":ti,ab,kw OR "Meditation, Mindfulness":ti,ab,kw OR "Mindfulness Meditations":ti,ab,kw | 25927 | 27727 |
| #16 | "mindfulness-based stress reduction"/exp | 1943 | 2118 |
| #17 | "Mindfulness-Based Stress Reduction":ti,ab,kw OR "Mindfulness Based Stress Reduction":ti,ab,kw OR "Mindfulness-Based Stress Reductions":ti,ab,kw OR "Stress Reduction, Mindfulness-Based":ti,ab,kw OR "MBSR Therapy":ti,ab,kw OR "MBSR Therapies":ti,ab,kw OR "Therapy, MBSR":ti,ab,kw OR "Mindfulness-Based Stress Reduction Therapy":ti,ab,kw OR "Mindfulness Based Stress Reduction Therapy":ti,ab,kw OR "MBSR":ti,ab,kw OR "mindfulness stress reduction course":ti,ab,kw OR "mindfulness stress reduction program":ti,ab,kw OR "mindfulness stress reduction training":ti,ab,kw OR "mindfulness stress reduction training program":ti,ab,kw OR "mindfulness-based stress reduction technique":ti,ab,kw OR "wellness-based mindfulness stress reduction intervention":ti,ab,kw OR "Mindfulness-Based":ti,ab,kw | 10128 | 10868 |
| #18 | "meditation"/exp | 14850 | 15444 |
| #19 | "Meditation":ti,ab,kw OR "Transcendental Meditation":ti,ab,kw OR "Meditation, Transcendental":ti,ab,kw | 13329 | 13929 |
| #20 | "yoga"/exp | 16135 | 17013 |
| #21 | "Yoga":ti,ab,kw OR "yogic meditation":ti,ab,kw | 13938 | 14762 |
| #22 | "stretching exercise"/exp | 7585 | 8091 |
| #23 | "Muscle Stretching Exercises":ti,ab,kw OR "Exercise, Muscle Stretching":ti,ab,kw OR "Muscle Stretching Exercise":ti,ab,kw OR "Active Stretching":ti,ab,kw OR "Stretching, Active":ti,ab,kw OR "Static-Active Stretching":ti,ab,kw OR "Static Active Stretching":ti,ab,kw OR "Stretching, Static-Active":ti,ab,kw OR "Proprioceptive Neuromuscular":ti,ab,kw OR "Facilitation (PNF) Stretching":ti,ab,kw OR "Proprioceptive Neuromuscular Facilitation":ti,ab,kw OR "Neuromuscular Facilitation, Proprioceptive":ti,ab,kw OR "Proprioceptive Neuromuscular Facilitations":ti,ab,kw OR "PNF Stretching":ti,ab,kw OR "PNF Stretchings":ti,ab,kw OR "Stretching, PNF":ti,ab,kw OR "PNF Stretching Exercise":ti,ab,kw OR "Exercise, PNF Stretching":ti,ab,kw OR "PNF Stretching Exercises":ti,ab,kw OR "Stretching Exercise, PNF":ti,ab,kw OR "Passive Stretching":ti,ab,kw OR "Stretching, Passive":ti,ab,kw OR "Static-Passive Stretching":ti,ab,kw OR "Static Passive Stretching":ti,ab,kw OR "Stretching, Static-Passive":ti,ab,kw OR "Relaxed Stretching":ti,ab,kw OR "Stretching, Relaxed":ti,ab,kw OR "Static Stretching":ti,ab,kw OR "Stretching, Static":ti,ab,kw OR "Isometric Stretching":ti,ab,kw OR "Stretching, Isometric":ti,ab,kw OR "Dynamic Stretching":ti,ab,kw OR "Stretching, Dynamic":ti,ab,kw OR "Ballistic Stretching":ti,ab,kw OR "Stretching, Ballistic":ti,ab,kw OR "stretching exercises":ti,ab,kw OR "stretching exercise":ti,ab,kw | 6057 | 6499 |
| #24 | "Tai Chi"/exp | 5357 | 5613 |
| #25 | "Tai Ji":ti,ab,kw OR "Tai-ji":ti,ab,kw OR "Tai Chi":ti,ab,kw OR "Chi, Tai":ti,ab,kw OR "Tai Chi Chuan":ti,ab,kw OR "Taiji":ti,ab,kw OR "Taijiquan":ti,ab,kw OR "T^ai Chi":ti,ab,kw OR "Tai Ji Quan":ti,ab,kw OR "Ji Quan, Tai":ti,ab,kw OR "Quan, Tai Ji":ti,ab,kw OR "Taiji quan":ti,ab,kw | 4881 | 5147 |
| #26 | "qigong"/exp | 1794 | 1897 |
| #27 | "Qigong":ti,ab,kw OR "Ch^i Kung":ti,ab,kw OR "Qi Gong":ti,ab,kw OR "chi kung":ti,ab,kw OR "chigung":ti,ab,kw | 2208 | 2312 |
| #28 | "dance therapy"/exp | 1153 | 1240 |
| #29 | "Dance Therapy":ti,ab,kw OR "Therapy, Dance":ti,ab,kw OR "Dance Therapies":ti,ab,kw OR "Therapies, Dance":ti,ab,kw OR "dance movement psychotherapy":ti,ab,kw OR "dance movement therapy":ti,ab,kw OR "dance psychotherapy":ti,ab,kw | 971 | 1031 |
| #30 | "movement therapy"/exp | 3834 | 3976 |
| #31 | "Exercise Therapy":ti,ab,kw OR "Exercise Prescription":ti,ab,kw OR "Rehabilitation Exercise":ti,ab,kw OR "Exercise, Rehabilitation":ti,ab,kw OR "Exercises, Rehabilitation":ti,ab,kw OR "Rehabilitation Exercises":ti,ab,kw OR "Remedial Exercise":ti,ab,kw OR "Exercise, Remedial":ti,ab,kw OR "Exercises, Remedial":ti,ab,kw OR "Remedial Exercises":ti,ab,kw OR "Therapy, Exercise":ti,ab,kw OR "Exercise Therapies":ti,ab,kw OR "Therapies, Exercise":ti,ab,kw OR "Physical Activity Intervention":ti,ab,kw OR "motion therapy":ti,ab,kw OR "movement-based therapy":ti,ab,kw OR "movement therapy":ti,ab,kw OR "Movement Therapies":ti,ab,kw OR "motion therapies":ti,ab,kw | 29496 | 31334 |
| #32 | "alternative medicine"/exp | 93128 | 95987 |
| #33 | "Mind-Body Therapies":ti,ab,kw OR "Mind Body Therapies":ti,ab,kw OR "Mind-Body Therapy":ti,ab,kw OR "Therapies, Mind-Body":ti,ab,kw OR "Therapy, Mind-Body":ti,ab,kw OR "Mind-Body Medicine":ti,ab,kw OR "Mind Body Medicine":ti,ab,kw OR "alternative therapies":ti,ab,kw OR "alternative therapy":ti,ab,kw OR "complementary medicine":ti,ab,kw OR "complementary therapies":ti,ab,kw OR "eclectic medicine":ti,ab,kw OR "eclecticism, historical":ti,ab,kw OR "historical eclecticism":ti,ab,kw OR "mental healing":ti,ab,kw OR "metaphysical mind-body relations":ti,ab,kw OR "mind body technique":ti,ab,kw OR "mind body therapy":ti,ab,kw OR "mind-body relations (metaphysics)":ti,ab,kw OR "mind-body relations, metaphysical":ti,ab,kw OR "polarity therapy":ti,ab,kw OR "radiaesthesia":ti,ab,kw OR "radiesthesia":ti,ab,kw OR "therapeutic cults":ti,ab,kw OR "Thomsonian medicine":ti,ab,kw OR "alternative medicine":ti,ab,kw OR "Mind-body exercise":ti,ab,kw | 52655 | 54320 |
| #34 | #4 OR #5 OR #6 OR #7 OR #8 OR #9 OR #10 OR #11 OR #12 OR #13 OR #14 OR #15 OR #16 OR #17 OR #18 OR #19 OR #20 OR #21 OR #22 OR #23 OR #24 OR #25 OR #26 OR #27 OR #28 OR #29 OR #30 OR #31 OR #32 OR #33 | 1035245 | 1066779 |
| #35 | #3 AND #34 | 9003 | 9390 |

**Table A4 Cochrane Central Register of Controlled Trials search strategy**

| **#** | **Search query** | **Records 10 Feb 2026** | **Records 27 Jul 2026** |
| --- | --- | --- | --- |
| #1 | [mh "Parkinson Disease"] | 6502 | 6685 |
| #2 | "Parkinson Disease":ti,ab,kw OR "Idiopathic Parkinson Disease":ti,ab,kw OR "Idiopathic Parkinson's Disease":ti,ab,kw OR "Lewy Body Parkinson Disease":ti,ab,kw OR "Lewy Body Parkinson's Disease":ti,ab,kw OR "Paralysis Agitans":ti,ab,kw OR "Parkinson Disease, Idiopathic":ti,ab,kw OR "Parkinson's Disease":ti,ab,kw OR "Parkinson's Disease, Idiopathic":ti,ab,kw OR "Parkinson's Disease, Lewy Body":ti,ab,kw OR "Primary Parkinsonism":ti,ab,kw OR "Parkinsonism, Primary":ti,ab,kw OR "idiopathic parkinsonism":ti,ab,kw OR "Lewy bodies of Parkinson disease":ti,ab,kw OR "Lewy bodies of Parkinson's disease":ti,ab,kw OR "Lewy bodies of Parkinsons disease":ti,ab,kw OR "Lewy body Parkinsons disease":ti,ab,kw OR "Parkinson dementia complex":ti,ab,kw OR "Parkinsons disease":ti,ab,kw | 14475 | 15018 |
| #3 | #1 OR #2 | 14475 | 15018 |
| #4 | [mh "Psychotherapy"] | 38297 | 39779 |
| #5 | "Psychotherapy":ti,ab,kw OR "Psychotherapies":ti,ab,kw OR "holistic psychotherapy":ti,ab,kw OR "multiple psychotherapy":ti,ab,kw OR "psychotherapeutic processes":ti,ab,kw OR "psychotherapeutic training":ti,ab,kw OR "psychotherapy, multiple":ti,ab,kw OR "socioenvironmental therapy":ti,ab,kw | 18043 | 18629 |
| #6 | [mh "Psychology, Clinical"] | 42 | 43 |
| #7 | "Psychology, Clinical":ti,ab,kw OR "Clinical Psychology":ti,ab,kw OR "clinical psychologist":ti,ab,kw OR "psychologist, clinical":ti,ab,kw | 1284 | 1334 |
| #8 | [mh "Acceptance and Commitment Therapy"] | 585 | 637 |
| #9 | "Acceptance and Commitment Therapy":ti,ab,kw OR "acceptance commitment therapy":ti,ab,kw OR "ACT psychotherapy":ti,ab,kw OR "ACT":ti,ab,kw OR "Psychological flexibility":ti,ab,kw OR "Acceptance-based":ti,ab,kw OR "Cognitive defusion":ti,ab,kw OR "Values-based":ti,ab,kw OR "Committed action":ti,ab,kw | 17213 | 18000 |
| #10 | ([mh "Cognitive Behavioral Therapy"] AND [mh "Mindfulness"]) OR [mh "Mindfulness-Based Cognitive Therapy"] | 2727 | 2933 |
| #11 | "Mindfulness-Based Cognitive Therapy":ti,ab,kw OR "Cognitive Therapy, Mindfulness-Based":ti,ab,kw OR "Mindfulness-Based Cognitive Therapies":ti,ab,kw OR "Mindfulness Based Cognitive Therapy":ti,ab,kw OR "Therapy, Mindfulness-Based Cognitive":ti,ab,kw OR "MBCT":ti,ab,kw OR "mindfulness cognitive therapy":ti,ab,kw OR "mindfulness-based CBT":ti,ab,kw OR "mindfulness-based cognitive behavior therapy":ti,ab,kw OR "mindfulness-based cognitive behavioral therapy":ti,ab,kw OR "mindfulness-based cognitive behavior therapy":ti,ab,kw OR "mindfulness-based cognitive behavioral therapy":ti,ab,kw OR "mindfulness-CBT":ti,ab,kw | 1231 | 1304 |
| #12 | "Cognitive Behavioral Therapy":ti,ab,kw OR "Behavioral Therapies, Cognitive":ti,ab,kw OR "Behavioral Therapy, Cognitive":ti,ab,kw OR "Cognitive Behavioral Therapies":ti,ab,kw OR "Therapies, Cognitive Behavioral":ti,ab,kw OR "Therapy, Cognitive Behavioral":ti,ab,kw OR "Cognition Therapy":ti,ab,kw OR "Cognition Therapies":ti,ab,kw OR "Therapies, Cognition":ti,ab,kw OR "Therapy, Cognitive Behavior":ti,ab,kw OR "Behavior Therapies, Cognitive":ti,ab,kw OR "Cognitive Behavior Therapies":ti,ab,kw OR "Therapies, Cognitive Behavior":ti,ab,kw OR "Therapy, Cognition":ti,ab,kw OR "Behavior Therapy, Cognitive":ti,ab,kw OR "Cognitive Behavior Therapy":ti,ab,kw OR "Cognitive Psychotherapy":ti,ab,kw OR "Cognitive Psychotherapies":ti,ab,kw OR "Psychotherapies, Cognitive":ti,ab,kw OR "Psychotherapy, Cognitive":ti,ab,kw OR "Therapy, Cognitive":ti,ab,kw OR "Cognitive Therapies":ti,ab,kw OR "Therapies, Cognitive":ti,ab,kw OR "Cognitive Behavior Therapy":ti,ab,kw OR "Behavior Therapies, Cognitive":ti,ab,kw OR "Behavior Therapy, Cognitive":ti,ab,kw OR "Cognitive Behavior Therapies":ti,ab,kw OR "Therapies, Cognitive Behavior":ti,ab,kw OR "Therapy, Cognitive Behavior":ti,ab,kw OR "Cognitive Therapy":ti,ab,kw OR "CBT":ti,ab,kw OR "cognitive behavior intervention":ti,ab,kw OR "cognitive behavior treatment":ti,ab,kw OR "cognitive behavioral intervention":ti,ab,kw OR "cognitive behavioral treatment":ti,ab,kw OR "cognitive behavior intervention":ti,ab,kw OR "cognitive behavior treatment":ti,ab,kw OR "cognitive behavioral intervention":ti,ab,kw OR "cognitive behavioral therapy":ti,ab,kw OR "cognitive behavioral treatment":ti,ab,kw OR "Third-wave CBT":ti,ab,kw | 31831 | 33049 |
| #13 | "Mindfulness":ti,ab,kw OR "Mindfulness Meditation":ti,ab,kw OR "Meditation, Mindfulness":ti,ab,kw OR "Mindfulness Meditations":ti,ab,kw | 12067 | 12928 |
| #14 | ([mh "Mindfulness"] AND [mh "Stress, Psychological"]) OR [mh "Mindfulness-Based Stress Reduction"] | 865 | 916 |
| #15 | "Mindfulness-Based Stress Reduction":ti,ab,kw OR "Mindfulness Based Stress Reduction":ti,ab,kw OR "Mindfulness-Based Stress Reductions":ti,ab,kw OR "Stress Reduction, Mindfulness-Based":ti,ab,kw OR "MBSR Therapy":ti,ab,kw OR "MBSR Therapies":ti,ab,kw OR "Therapy, MBSR":ti,ab,kw OR "Mindfulness-Based Stress Reduction Therapy":ti,ab,kw OR "Mindfulness Based Stress Reduction Therapy":ti,ab,kw OR "MBSR":ti,ab,kw OR "mindfulness stress reduction course":ti,ab,kw OR "mindfulness stress reduction program":ti,ab,kw OR "mindfulness stress reduction training":ti,ab,kw OR "mindfulness stress reduction training program":ti,ab,kw OR "mindfulness-based stress reduction technique":ti,ab,kw OR "wellness-based mindfulness stress reduction intervention":ti,ab,kw OR "Mindfulness-Based":ti,ab,kw | 5586 | 5987 |
| #16 | [mh "Meditation"] | 1167 | 1213 |
| #17 | "Meditation":ti,ab,kw OR "Transcendental Meditation":ti,ab,kw OR "Meditation, Transcendental":ti,ab,kw | 5623 | 5976 |
| #18 | [mh "Yoga"] | 1325 | 1403 |
| #19 | "Yoga":ti,ab,kw OR "yogic meditation":ti,ab,kw | 7091 | 7616 |
| #20 | [mh "Muscle Stretching Exercises"] | 974 | 1031 |
| #21 | "Muscle Stretching Exercises":ti,ab,kw OR "Exercise, Muscle Stretching":ti,ab,kw OR "Muscle Stretching Exercise":ti,ab,kw OR "Active Stretching":ti,ab,kw OR "Stretching, Active":ti,ab,kw OR "Static-Active Stretching":ti,ab,kw OR "Static Active Stretching":ti,ab,kw OR "Stretching, Static-Active":ti,ab,kw OR "Proprioceptive Neuromuscular":ti,ab,kw OR "Facilitation (PNF) Stretching":ti,ab,kw OR "Proprioceptive Neuromuscular Facilitation":ti,ab,kw OR "Neuromuscular Facilitation, Proprioceptive":ti,ab,kw OR "Proprioceptive Neuromuscular Facilitations":ti,ab,kw OR "PNF Stretching":ti,ab,kw OR "PNF Stretchings":ti,ab,kw OR "Stretching, PNF":ti,ab,kw OR "PNF Stretching Exercise":ti,ab,kw OR "Exercise, PNF Stretching":ti,ab,kw OR "PNF Stretching Exercises":ti,ab,kw OR "Stretching Exercise, PNF":ti,ab,kw OR "Passive Stretching":ti,ab,kw OR "Stretching, Passive":ti,ab,kw OR "Static-Passive Stretching":ti,ab,kw OR "Static Passive Stretching":ti,ab,kw OR "Stretching, Static-Passive":ti,ab,kw OR "Relaxed Stretching":ti,ab,kw OR "Stretching, Relaxed":ti,ab,kw OR "Static Stretching":ti,ab,kw OR "Stretching, Static":ti,ab,kw OR "Isometric Stretching":ti,ab,kw OR "Stretching, Isometric":ti,ab,kw OR "Dynamic Stretching":ti,ab,kw OR "Stretching, Dynamic":ti,ab,kw OR "Ballistic Stretching":ti,ab,kw OR "Stretching, Ballistic":ti,ab,kw OR "stretching exercises":ti,ab,kw OR "stretching exercise":ti,ab,kw | 5493 | 5863 |
| #22 | [mh "Tai Ji"] | 642 | 681 |
| #23 | "Tai Ji":ti,ab,kw OR "Tai-ji":ti,ab,kw OR "Tai Chi":ti,ab,kw OR "Chi, Tai":ti,ab,kw OR "Tai Chi Chuan":ti,ab,kw OR "Taiji":ti,ab,kw OR "Taijiquan":ti,ab,kw OR "T'ai Chi":ti,ab,kw OR "Tai Ji Quan":ti,ab,kw OR "Ji Quan, Tai":ti,ab,kw OR "Quan, Tai Ji":ti,ab,kw OR "Taiji quan":ti,ab,kw | 2300 | 2417 |
| #24 | [mh "Qigong"] | 198 | 205 |
| #25 | "Qigong":ti,ab,kw OR "Ch'i Kung":ti,ab,kw OR "Qi Gong":ti,ab,kw OR "chi kung":ti,ab,kw OR "chigung":ti,ab,kw | 965 | 1021 |
| #26 | [mh "Dance Therapy"] | 150 | 161 |
| #27 | "Dance Therapy":ti,ab,kw OR "Therapy, Dance":ti,ab,kw OR "Dance Therapies":ti,ab,kw OR "Therapies, Dance":ti,ab,kw OR "dance movement psychotherapy":ti,ab,kw OR "dance movement therapy":ti,ab,kw OR "dance psychotherapy":ti,ab,kw | 356 | 379 |
| #28 | [mh "Exercise Therapy"] | 24199 | 25327 |
| #29 | "Exercise Therapy":ti,ab,kw OR "Exercise Prescription":ti,ab,kw OR "Rehabilitation Exercise":ti,ab,kw OR "Exercise, Rehabilitation":ti,ab,kw OR "Exercises, Rehabilitation":ti,ab,kw OR "Rehabilitation Exercises":ti,ab,kw OR "Remedial Exercise":ti,ab,kw OR "Exercise, Remedial":ti,ab,kw OR "Exercises, Remedial":ti,ab,kw OR "Remedial Exercises":ti,ab,kw OR "Therapy, Exercise":ti,ab,kw OR "Exercise Therapies":ti,ab,kw OR "Therapies, Exercise":ti,ab,kw OR "Physical Activity Intervention":ti,ab,kw OR "motion therapy":ti,ab,kw OR "movement-based therapy":ti,ab,kw OR "movement therapy":ti,ab,kw OR "Movement Therapies":ti,ab,kw OR "motion therapies":ti,ab,kw | 29426 | 30752 |
| #30 | [mh "Mind-Body Therapies"] | 10297 | 10792 |
| #31 | "Mind-Body Therapies":ti,ab,kw OR "Mind Body Therapies":ti,ab,kw OR "Mind-Body Therapy":ti,ab,kw OR "Therapies, Mind-Body":ti,ab,kw OR "Therapy, Mind-Body":ti,ab,kw OR "Mind-Body Medicine":ti,ab,kw OR "Mind Body Medicine":ti,ab,kw OR "alternative therapies":ti,ab,kw OR "alternative therapy":ti,ab,kw OR "complementary medicine":ti,ab,kw OR "complementary therapies":ti,ab,kw OR "eclectic medicine":ti,ab,kw OR "eclecticism, historical":ti,ab,kw OR "historical eclecticism":ti,ab,kw OR "mental healing":ti,ab,kw OR "metaphysical mind-body relations":ti,ab,kw OR "mind body technique":ti,ab,kw OR "mind body therapy":ti,ab,kw OR "mind-body relations (metaphysics)":ti,ab,kw OR "mind-body relations, metaphysical":ti,ab,kw OR "polarity therapy":ti,ab,kw OR "radiaesthesia":ti,ab,kw OR "radiesthesia":ti,ab,kw OR "therapeutic cults":ti,ab,kw OR "Thomsonian medicine":ti,ab,kw OR "alternative medicine":ti,ab,kw OR "Mind-body exercise":ti,ab,kw | 6380 | 6941 |
| #32 | #4 OR #5 OR #6 OR #7 OR #8 OR #9 OR #10 OR #11 OR #12 OR #13 OR #14 OR #15 OR #16 OR #17 OR #18 OR #19 OR #20 OR #21 OR #22 OR #23 OR #24 OR #25 OR #26 OR #27 OR #28 OR #29 OR #30 OR #31 | 137564 | 144365 |
| #33 | #3 AND #32 | 1389 | 1474 |

**Table A5 Web of Science search strategy**

| **#** | **Search query** | **Records 27 Jul 2026** |
| --- | --- | --- |
| #1 | TS=("Parkinson Disease" OR "Idiopathic Parkinson Disease" OR "Idiopathic Parkinson's Disease" OR "Lewy Body Parkinson Disease" OR "Lewy Body Parkinson's Disease" OR "Paralysis Agitans" OR "Parkinson Disease, Idiopathic" OR "Parkinson's Disease" OR "Parkinson's Disease, Idiopathic" OR "Parkinson's Disease, Lewy Body" OR "Primary Parkinsonism" OR "Parkinsonism, Primary" OR "idiopathic parkinsonism" OR "Lewy bodies of Parkinson disease" OR "Lewy bodies of Parkinson's disease" OR "Lewy bodies of Parkinsons disease" OR "Lewy body Parkinsons disease" OR "Parkinson dementia complex" OR "Parkinsons disease") | 202324 |
| #2 | TS=("Psychotherapy" OR "Psychotherapies" OR "holistic psychotherapy" OR "multiple psychotherapy" OR "psychotherapeutic processes" OR "psychotherapeutic training" OR "psychotherapy, multiple" OR "socioenvironmental therapy") | 52804 |
| #3 | TS=("Psychology, Clinical" OR "Clinical Psychology" OR "clinical psychologist" OR "psychologist, clinical") | 4191 |
| #4 | TS=("Acceptance and Commitment Therapy" OR "acceptance commitment therapy" OR "ACT psychotherapy" OR "ACT" OR "Psychological flexibility" OR "Acceptance-based" OR "Cognitive defusion" OR "Values-based" OR "Committed action") | 509911 |
| #5 | TS=("Mindfulness-Based Cognitive Therapy" OR "Cognitive Therapy, Mindfulness-Based" OR "Mindfulness-Based Cognitive Therapies" OR "Mindfulness Based Cognitive Therapy" OR "Therapy, Mindfulness-Based Cognitive" OR "MBCT" OR "mindfulness cognitive therapy" OR "mindfulness-based CBT" OR "mindfulness-based cognitive behavior therapy" OR "mindfulness-based cognitive behavioral therapy" OR "mindfulness-based cognitive behavior therapy" OR "mindfulness-based cognitive behavioral therapy" OR "mindfulness-CBT") | 1519 |
| #6 | TS=("Cognitive Behavioral Therapy" OR "Behavioral Therapies, Cognitive" OR "Behavioral Therapy, Cognitive" OR "Cognitive Behavioral Therapies" OR "Therapies, Cognitive Behavioral" OR "Therapy, Cognitive Behavioral" OR "Cognition Therapy" OR "Cognition Therapies" OR "Therapies, Cognition" OR "Therapy, Cognitive Behavior" OR "Behavior Therapies, Cognitive" OR "Cognitive Behavior Therapies" OR "Therapies, Cognitive Behavior" OR "Therapy, Cognition" OR "Behavior Therapy, Cognitive" OR "Cognitive Behavior Therapy" OR "Cognitive Psychotherapy" OR "Cognitive Psychotherapies" OR "Psychotherapies, Cognitive" OR "Psychotherapy, Cognitive" OR "Therapy, Cognitive" OR "Cognitive Therapies" OR "Therapies, Cognitive" OR "Cognitive Behavior Therapy" OR "Behavior Therapies, Cognitive" OR "Behavior Therapy, Cognitive" OR "Cognitive Behavior Therapies" OR "Therapies, Cognitive Behavior" OR "Therapy, Cognitive Behavior" OR "Cognitive Therapy" OR "CBT" OR "cognitive behavior intervention" OR "cognitive behavior treatment" OR "cognitive behavioral intervention" OR "cognitive behavioral treatment" OR "cognitive behavior intervention" OR "cognitive behavior treatment" OR "cognitive behavioral intervention" OR "cognitive behavioral therapy" OR "cognitive behavioral treatment" OR "Third-wave CBT") | 61298 |
| #7 | TS=("Mindfulness" OR "Mindfulness Meditation" OR "Meditation, Mindfulness" OR "Mindfulness Meditations") | 31680 |
| #8 | TS=("Mindfulness-Based Stress Reduction" OR "Mindfulness Based Stress Reduction" OR "Mindfulness-Based Stress Reductions" OR "Stress Reduction, Mindfulness-Based" OR "MBSR Therapy" OR "MBSR Therapies" OR "Therapy, MBSR" OR "Mindfulness-Based Stress Reduction Therapy" OR "Mindfulness Based Stress Reduction Therapy" OR "MBSR" OR "mindfulness stress reduction course" OR "mindfulness stress reduction program" OR "mindfulness stress reduction training" OR "mindfulness stress reduction training program" OR "mindfulness-based stress reduction technique" OR "wellness-based mindfulness stress reduction intervention" OR "Mindfulness-Based") | 9436 |
| #9 | TS=("Meditation" OR "Transcendental Meditation" OR "Meditation, Transcendental") | 13721 |
| #10 | TS=("Yoga" OR "yogic meditation") | 8916 |
| #11 | TS=("Muscle Stretching Exercises" OR "Exercise, Muscle Stretching" OR "Muscle Stretching Exercise" OR "Active Stretching" OR "Stretching, Active" OR "Static-Active Stretching" OR "Static Active Stretching" OR "Stretching, Static-Active" OR "Proprioceptive Neuromuscular" OR "Facilitation (PNF) Stretching" OR "Proprioceptive Neuromuscular Facilitation" OR "Neuromuscular Facilitation, Proprioceptive" OR "Proprioceptive Neuromuscular Facilitations" OR "PNF Stretching" OR "PNF Stretchings" OR "Stretching, PNF" OR "PNF Stretching Exercise" OR "Exercise, PNF Stretching" OR "PNF Stretching Exercises" OR "Stretching Exercise, PNF" OR "Passive Stretching" OR "Stretching, Passive" OR "Static-Passive Stretching" OR "Static Passive Stretching" OR "Stretching, Static-Passive" OR "Relaxed Stretching" OR "Stretching, Relaxed" OR "Static Stretching" OR "Stretching, Static" OR "Isometric Stretching" OR "Stretching, Isometric" OR "Dynamic Stretching" OR "Stretching, Dynamic" OR "Ballistic Stretching" OR "Stretching, Ballistic" OR "stretching exercises" OR "stretching exercise") | 3732 |
| #12 | TS=("Tai Ji" OR "Tai-ji" OR "Tai Chi" OR "Chi, Tai" OR "Tai Chi Chuan" OR "Taiji" OR "Taijiquan" OR "T'ai Chi" OR "Tai Ji Quan" OR "Ji Quan, Tai" OR "Quan, Tai Ji" OR "Taiji quan") | 5588 |
| #13 | TS=("Qigong" OR "Ch'i Kung" OR "Qi Gong" OR "chi kung" OR "chigung") | 1769 |
| #14 | TS=("Dance Therapy" OR "Therapy, Dance" OR "Dance Therapies" OR "Therapies, Dance" OR "dance movement psychotherapy" OR "dance movement therapy" OR "dance psychotherapy") | 700 |
| #15 | TS=("Exercise Therapy" OR "Exercise Prescription" OR "Rehabilitation Exercise" OR "Exercise, Rehabilitation" OR "Exercises, Rehabilitation" OR "Rehabilitation Exercises" OR "Remedial Exercise" OR "Exercise, Remedial" OR "Exercises, Remedial" OR "Remedial Exercises" OR "Therapy, Exercise" OR "Exercise Therapies" OR "Therapies, Exercise" OR "Physical Activity Intervention" OR "motion therapy" OR "movement-based therapy" OR "movement therapy" OR "Movement Therapies" OR "motion therapies") | 21676 |
| #16 | TS=("Mind-Body Therapies" OR "Mind Body Therapies" OR "Mind-Body Therapy" OR "Therapies, Mind-Body" OR "Therapy, Mind-Body" OR "Mind-Body Medicine" OR "Mind Body Medicine" OR "alternative therapies" OR "alternative therapy" OR "complementary medicine" OR "complementary therapies" OR "eclectic medicine" OR "eclecticism, historical" OR "historical eclecticism" OR "mental healing" OR "metaphysical mind-body relations" OR "mind body technique" OR "mind body therapy" OR "mind-body relations (metaphysics)" OR "mind-body relations, metaphysical" OR "polarity therapy" OR "radiaesthesia" OR "radiesthesia" OR "therapeutic cults" OR "Thomsonian medicine" OR "alternative medicine" OR "Mind-body exercise") | 36103 |
| #17 | #2 OR #3 OR #4 OR #5 OR #6 OR #7 OR #8 OR #9 OR #10 OR #11 OR #12 OR #13 OR #14 OR #15 OR #16 | 709638 |
| #18 | #1 AND #17 | 4184 |

Web of Science was searched using topic-field (TS) queries, as shown above. This database was added following peer review and was therefore searched only on 27 July 2026.

**Supplementary Table S1 Selected reports requiring individual explanation during eligibility assessment, with reasons for non-inclusion**

| **Report** | **Identified from** | **Reason for exclusion** | **Basis** |
| --- | --- | --- | --- |
| ***Studies included in Yu et al. (2025) for the anxiety outcome*** |  |  |  |
| Calleo et al. (2015) | Yu et al. (2025) | Not a randomized controlled trial | The authors state that, following a misassignment error, the study is referred to as a controlled trial rather than a randomized controlled trial. Eligibility was restricted to randomized controlled trials. |
| Hadinia et al. (2016) | Yu et al. (2025) | No validated anxiety outcome | The outcomes reported are the PDQ-39, BELA and FKK, none of which is a validated anxiety measure. |
| Lizhen et al. (2022) | Yu et al. (2025) | Not retrievable within the search scope | Retrievable only through Chinese-language databases, which were not among the databases searched for this review. |
| Rodgers et al. (2019) | Yu et al. (2025) | Effect estimate not derivable | Eligible in design, population and outcome. The reported dispersion statistics cannot be interpreted unambiguously as standard deviations of the observed scores, and taking them at face value yields an estimate irreconcilable with the trial’s own reported result. |
| Xiaohong et al. (2017) | Yu et al. (2025) | Not retrievable within the search scope | Retrievable only through Chinese-language databases (CNKI, Wanfang, VIP, CBM), which were not among the databases searched for this review. |
| ***Reports identified in the updated search (27 July 2026)*** |  |  |  |
| Li et al. (2022) | Updated search | Intervention outside scope; dispersion values inconsistent | Health qigong, which does not state a cognitive, acceptance-based or mindfulness-based mechanism. The reported dispersion values are additionally inconsistent across the report. |
| Pinto et al. (2025) (PACT) | Updated search | Anxiety data not extractable | Group-level means and standard deviations for the GAD-7 are not reported. |
| Sun et al. (2026) | Updated search | Anxiety data not extractable | Only model-adjusted means and standard errors from generalized estimating equations are reported. |
| Volpe et al. (2025) | Updated search | Anxiety data not extractable | Improvement rates only are reported; group-level means and standard deviations for the State–Trait Anxiety Inventory are not available. |
| Wang et al. (2023) | Updated search | Intervention outside scope; not published in English | Tai chi, which does not state a cognitive, acceptance-based or mindfulness-based mechanism. |

*Note. This table lists reports that met the design, population and intervention criteria on initial assessment, or that were identified as included by a previous overlapping review, and that were subsequently excluded. It is not a list of all 125 reports excluded at full text; the complete count and the distribution of exclusion reasons are given in Figure 1. Reports are ordered alphabetically within each group. Dobkin et al. (2011) and Okai et al. (2013), which Yu et al. (2025) list for the anxiety outcome, are included in the present synthesis, as is Bogosian et al. (2022), which is listed in neither review.*

**Supplementary Table S2 Results of the leave-one-out sensitivity analysis**

| **Omitted study** | **Pooled Hedges’ g** | **95% confidence interval** | **I² (%)** |
| --- | --- | --- | --- |
| Bogosian 2022 | −0.53 | [−0.68, −0.37] | 0.0 |
| Buchwitz 2021 | −0.47 | [−0.69, −0.25] | 44.6 |
| Dobkin 2011 | −0.43 | [−0.65, −0.21] | 39.9 |
| Dobkin 2020 | −0.43 | [−0.65, −0.21] | 40.5 |
| Dobkin 2021 | −0.44 | [−0.66, −0.21] | 42.8 |
| Koszycki 2025 | −0.50 | [−0.71, −0.30] | 34.1 |
| Kraepelien 2020 | −0.45 | [−0.68, −0.22] | 45.6 |
| Kwok 2019 | −0.48 | [−0.71, −0.24] | 43.8 |
| Kwok 2025 | −0.44 | [−0.67, −0.21] | 42.7 |
| Moonen 2021 | −0.46 | [−0.69, −0.24] | 45.6 |
| Okai 2013 | −0.44 | [−0.65, −0.22] | 42.0 |
| Troeung 2014 | −0.46 | [−0.68, −0.23] | 45.5 |
| Wuthrich & Rapee 2019 | −0.46 | [−0.68, −0.24] | 45.6 |

*Note. Each row presents the pooled random-effects estimate (Hedges’ g, restricted maximum likelihood estimation with the Hartung–Knapp adjustment) after omitting the indicated study. Trials are ordered alphabetically by first author. The pooled estimate remained statistically significant across all iterations, ranging from −0.53 to −0.43. Omission of Bogosian et al. (2022) reduced I² from 40.7% to 0%.*

**Supplementary Table S3 Comparison of between-study variance estimators with Hartung–Knapp inference and prediction intervals**

| **Estimator of τ²** | **τ²** | **Hedges’ g [95% CI], Hartung–Knapp** | **95% prediction interval** |
| --- | --- | --- | --- |
| REML (primary) | 0.052 | −0.46 [−0.67, −0.25] | −1.00 to +0.08 |
| Paule–Mandel | 0.044 | −0.46 [−0.67, −0.25] | −0.96 to +0.04 |
| DerSimonian–Laird | 0.045 | −0.46 [−0.67, −0.25] | −0.96 to +0.04 |

*Note. Restricted maximum likelihood was used as the primary estimator of between-study variance, with inference based on the Hartung–Knapp adjustment. The three estimators yielded an identical pooled estimate to two decimal places. The 95% prediction interval included the null under every specification.*

**Supplementary Table S4 Sensitivity analyses by type of anxiety instrument**

| **Analysis** | **k** | **N** | **Hedges’ g** | **95% confidence interval** | **I² (%)** |
| --- | --- | --- | --- | --- | --- |
| **All included trials** | **13** | **877** | **−0.46** | **[−0.67, −0.25]** | **40.7** |
| Clinician-administered instruments | 4 | 290 | −0.68 | [−0.92, −0.45] | 0.0 |
| Self-report instruments | 9 | 587 | −0.35 | [−0.63, −0.06] | 44.1 |
| Excluding the STAI-T trial | 12 | 847 | −0.47 | [−0.69, −0.25] | 44.6 |
| Instruments with substantial somatic content | 7 | 402 | −0.58 | [−0.87, −0.30] | 21.4 |
| Instruments with minimal somatic content | 6 | 475 | −0.33 | [−0.70, 0.05] | 50.7 |
| HADS-A only | 4 | 434 | −0.31 | [−0.96, 0.34] | 69.7 |
| HAM-A only | 3 | 242 | −0.74 | [−0.89, −0.59] | 0.0 |

*Note. Instruments were classified as having substantial somatic content (Hamilton Anxiety Rating Scale, Beck Anxiety Inventory, Depression Anxiety Stress Scales–Anxiety) or minimal somatic content (Hospital Anxiety and Depression Scale–Anxiety, Geriatric Anxiety Inventory, State–Trait Anxiety Inventory–Trait). Neither subgroup contrast was statistically significant (instrument administration: Qb = 2.54, df = 1, p = .111; somatic content: Qb = 1.64, df = 1, p = .200). These analyses were not prespecified and are exploratory. Instrument type is confounded with intervention category, blinding of outcome assessment and risk of bias.*

**Supplementary Table S5 Sensitivity analysis by risk of bias**

| **Analysis** | **k** | **N** | **Hedges’ g** | **95% confidence interval** | **I² (%)** | **95% prediction interval** |
| --- | --- | --- | --- | --- | --- | --- |
| **All included trials** | **13** | **877** | **−0.46** | **[−0.67, −0.25]** | **40.7** | **[−1.00, 0.08]** |
| Excluding trials at high risk of bias | 7 | 650 | −0.52 | [−0.77, −0.28] | 30.2 | [−0.93, −0.12] |
| Restricted to trials at low risk of bias | 5 | 353 | −0.55 | [−0.95, −0.14] | 42.3 | [−1.26, 0.16] |

*Note. Overall risk-of-bias judgments are presented in Figure 2: five trials were at low risk, two raised some concerns, and six were at high risk. Exclusion of trials at high risk of bias was specified in the original analytical plan. The trials at low risk of bias are not a random subset of the evidence base: all five evaluated cognitive–behavioral or interpersonal interventions.*

**Supplementary Table S6 Trial-level effect estimates at follow-up, with the summary statistics reported by each trial**

| **Trial** | **Follow-up** | **Outcome** | **Intervention group (n; reported statistic)** | **Control group (n; reported statistic)** | **Data transformation** | **Hedges' g (95% CI)** | **Control-group status before follow-up** |
| --- | --- | --- | --- | --- | --- | --- | --- |
| Bogosian et al. (2022) | 12 weeks | HADS-A | 30; mean 6.97 (SD 4.44) | 30; mean 6.17 (SD 3.87) | None | +0.19 (−0.32 to +0.70) | Waiting list; the intervention was offered after the 3-month follow-up |
| Buchwitz et al. (2021) | 8 weeks | STAI-T | 14; median 35.5 (range 24–56) | 16; median 35.0 (range 26–75) | Mean and SD estimated from median and range (Wan et al.), giving 37.75 (SD 9.37) and 42.75 (SD 13.85); the published analysis carried the last observation forward for three missing follow-up values, so the post-assessment sample of 14 and 16 was used | −0.41 (−1.13 to +0.32) | Waiting list; the intervention was offered after the end of the study |
| Dobkin et al. (2011) | 14 weeks | HAM-A | 41; mean 15.36 (SD 4.60) | 39; mean 18.30 (SD 4.43) | None | −0.64 (−1.09 to −0.20) | Clinical monitoring; cognitive behavioral therapy became available only after the 14-week assessment |
| Dobkin et al. (2020) | 6 months | HAM-A | 37; mean 16.51 (SD 4.43) | 35; mean 18.91 (SD 4.50) | None | −0.53 (−1.00 to −0.06) | Treatment as usual; monthly booster sessions were permitted in the intervention arm during the extension phase |
| Dobkin et al. (2021) | 6 months | HAM-A | 45; mean 20.41 (SD 4.61) | 45; mean 23.65 (SD 5.17) | None | −0.66 (−1.08 to −0.23) | Treatment as usual; monthly booster sessions were permitted in the intervention arm during the extension phase |
| Koszycki et al. (2025) | 6 months | BAI | 32; adjusted mean 15.74 (SE 2.0) | 31; adjusted mean 18.12 (SE 2.0) | SD approximated as SE × √n | −0.21 (−0.71 to +0.29) | Active comparator (supportive therapy) maintained; no crossover to the experimental intervention |
| Kwok et al. (2019) | 3 months (week 20) | HADS-A | 57; mean 3.04 (SD 3.06) | 55; mean 4.95 (SD 3.49) | None. The reported means and standard deviations are descriptive statistics for participants assessed at the 20-week follow-up, so the numbers assessed at that time point were used | −0.58 (−0.96 to −0.20) | Active comparator (stretching and resistance training) maintained; no crossover |
| Kwok et al. (2025) | 6 months | HADS-A | 105 (meditation 53, yoga 52); adjusted means 5.22 (95% CI 4.55–5.89) and 5.61 (95% CI 4.98–6.23) | 54; adjusted mean 5.90 (95% CI 5.26–6.54) | Arms combined at the group-data level; SD approximated from the reported confidence intervals | −0.20 (−0.53 to +0.13) | Waiting-list usual care; the mindfulness programs were offered after the end of the 6-month study |
| Moonen et al. (2021) | 3 months | HAM-A | 21; mean 11.70 (SD 5.80) | 21; mean 14.10 (SD 7.20) | None | −0.36 (−0.97 to +0.25) | Clinical monitoring; cognitive behavioral therapy became available only after the 3-month assessment |
| **Pooled (k = 9; n = 708)** |  |  |  |  |  | **−0.39 (−0.60 to −0.18)** | **I² = 26.0%** |
| **Excluding the 8-week follow-up (k = 8; n = 678)** |  |  |  |  |  | **−0.39 (−0.62 to −0.15)** | **I² = 35.4%** |
| **Restricted to 6-month follow-up (k = 4; n = 384)** |  |  |  |  |  | **−0.38 (−0.75 to −0.02)** | **I² = 16.7%** |
| **Excluding Kwok et al. (2025) (k = 8; n = 549)** |  |  |  |  |  | **−0.43 (−0.67 to −0.19)** | **I² = 24.6%** |

Follow-up data are presented in the form reported by each trial. Means and standard deviations were used where available; adjusted means with standard errors or 95% confidence intervals, and medians with ranges, are identified explicitly. Where necessary, standard deviations were approximated from reported standard errors or confidence intervals, and means and standard deviations were estimated from medians and ranges using the method of Wan et al. Estimates were pooled using restricted maximum likelihood estimation with the Hartung–Knapp adjustment. Follow-up estimates were included where both randomized groups were assessed after the intervention period and the control group had not crossed over to the experimental intervention before the follow-up assessment; two trials used an active comparator, which was maintained throughout. In Dobkin et al. (2020) and Dobkin et al. (2021), monthly booster sessions were permitted in the intervention arm during the extension phase, so these estimates represent effects observed at post-treatment follow-up or during an extension phase rather than maintenance after complete treatment cessation. For Kwok et al. (2025), values are linear mixed-model adjusted means with 95% confidence intervals; because observed and residual standard deviations were not reported, standard errors were derived from the confidence intervals and approximate standard deviations were calculated as SE × √n. The resulting arm-level standardized effects (0.565 for meditation and 0.683 for yoga) were closely consistent with the Cohen’s d values reported by the original authors (0.57 and 0.69). Excluding this trial did not materially change the pooled follow-up estimate. Four trials could not contribute a follow-up estimate: in Kraepelien et al. (2020) and Troeung et al. (2014) the control condition received the intervention immediately after the post-treatment assessment; in Okai et al. (2013) the six-month assessment was the trial endpoint rather than a follow-up; and in Wuthrich and Rapee (2019) follow-up values were not reported for both groups.

**Supplementary Table S7 Pooled estimate under alternative methods of handling the three-arm trial**

| **Specification** | **k** | **Hedges’ g [95% CI]** | **Note** |
| --- | --- | --- | --- |
| Arms combined at group level (adopted primary analysis) | 13 | −0.46 [−0.67, −0.25] | I² = 40.7%; no assumption about the correlation between arm-level estimates is required |
| Shared control group divided | 14 | −0.47 [−0.66, −0.27] | I² = 36.1%; control group divided equally between the two comparisons |
| Robust variance estimation | 14 | −0.46 [−0.68, −0.25] | Correlated-effects working model with ρ = 0.5; CR2 small-sample correction; df = 11.0 |
| Three-level random-effects model | 14 | −0.46 [−0.67, −0.24] | τ² at trial level = 0.052; τ² at comparison level = 0.000 |

*Note. Kwok et al. (2025) randomized participants to meditation, yoga or usual care. In the primary analysis the two active arms were combined at the group-data level, giving a single comparison of 105 intervention against 54 control participants. Arm-level post-treatment values were derived from the adjusted means and 95% confidence intervals reported in the source, with standard deviations obtained as SE × √n; the resulting arm-level effect sizes correspond closely to the Cohen’s d values reported by the original authors. The pooled estimate is unchanged to two decimal places under three of the four specifications, and the comparison-level variance component in the three-level model was estimated at zero.*
